# Landscape Features and Climatic Forces Shape the Genetic Structure and Evolutionary History of an Oak Species (*Quercus chenii*) in East China

**DOI:** 10.3389/fpls.2019.01060

**Published:** 2019-09-03

**Authors:** Yao Li, Xingwang Zhang, Yanming Fang

**Affiliations:** ^1^Co-Innovation Center for Sustainable Forestry in Southern China, College of Biology and the Environment, Key Laboratory of State Forestry and Grassland Administration on Subtropical Forest Biodiversity Conservation, Nanjing Forestry University, Nanjing, China; ^2^College of Life Sciences, Huaibei Normal University, Huaibei, China

**Keywords:** chloroplast haplotypes, East China, elevation, environmental isolation, geographic isolation, landscape features, microsatellites, *Quercus chenii*

## Abstract

Major topographic features facilitate intraspecific divergence through geographic isolation. This process may be enhanced by environmental isolation along climatic gradients, but also may be reduced by range shifts under rapid climatic changes. In this study, we examined how topography and climate have interacted over time and space to influence the genetic structure and evolutionary history of *Quercus chenii*, a deciduous oak species representative of the East China flora. Based on the nuclear microsatellite variation at 14 loci, we identified multiple genetic boundaries that were well associated with persistent landscape barriers of East China. Redundancy analysis indicated that both geography and climate explained similar amounts of intraspecific variation. Ecological differences along altitudinal gradients may have driven the divergence between highlands and lowlands. However, range expansions during the Last Interglacial as inferred from approximate Bayesian computation (ABC) may have increased the genetic diversity and eliminated the differentiation of lowland populations *via* admixture. Chloroplast (cp) DNA analysis of four intergenic spacers (2,866 bp in length) identified a total of 18 haplotypes, 15 of which were private to a single population, probably a result of long-term isolation among multiple montane habitats. A time-calibrated phylogeny suggested that palaeoclimatic changes of the Miocene underlay the lineage divergence of three major clades. In combination with ecological niche modeling (ENM), we concluded that mountainous areas with higher climatic stability are more likely to be glacial refugia that preserved higher phylogenetic diversity, while plains and basins may have acted as dispersal corridors for the post-glacial south-to-north migration. Our findings provide compelling evidence that both topography and climate have shaped the pattern of genetic variation of *Q. chenii*. Mountains as barriers facilitated differentiation through both geographic and environmental isolation, whereas lowlands as corridors increased the population connectivity especially when the species experienced range expansions.

## Introduction

Tectonically driven uplift and climatically driven erosion create high-elevation mountains and deep river valleys upon the earth (Badgley et al., 2017). Interactions of these landscape features with climate forces affect the local patterns of intraspecific genetic variation in different ways. For example, along horizontal axes, mountain ridges may serve as either barriers or corridors for range shifts in response to global warming, depending on the species’ altitudinal distribution (Ohsawa and Ide, 2008; Tian et al., 2018). Along vertical axes, mountains may increase the dissimilarity of microclimatic conditions between highlands and lowlands, and further facilitate population differentiation through the interactive effects of both non-adaptive (i.e., genetic drift and gene flow) and adaptive (i.e., natural selection) evolutionary processes across environmental gradients (Gonzalo-Turpin and Hazard, 2009; Chen et al., 2014; De Villemereuil et al., 2018).

Interactions of topography and climate are responsible for the patterns of genetic variation at a species’ level. The occurrence of high genetic diversity in mountainous areas may be attributed to the long-term *in situ* diversification among isolated habitats (Qu et al., 2014; Valderrama et al., 2014; Meng et al., 2017). Mountain ranges are also characterized by lower climate change velocity and great habitat heterogeneity, which would contribute to the survival of both immigrants and residents in refugia with ecologically stable habitats (Sandel et al., 2011; Gavin et al., 2014; Harrison and Noss, 2017). By contrast, populations of less mountainous areas, such as lowlands, are more likely to be influenced by climatic fluctuations. The levels of genetic diversity preserved after a climate change not only depend on the dispersal abilities of a species, but also on the speed of the change (Arenas et al., 2011). Nonetheless, low climatic stability may also result in high levels of genetic diversity and admixture, due to recurrent post-glacial colonization by individuals from genetically differentiated populations (Petit et al., 2003; Ortego et al., 2015).

The East China Floristic Province is one of the richest regions of plant diversity in the Sino-Japanese Floristic Region (Wu, 1979). The topography here is characterized by numerous plains and basins interspersed between low hills and median-high mountains (Cao et al., 2018). Those ranges, with a group of peaks exceeding 1,500 m in elevation, are scattered from the northeast (e.g., the Tianmu Mountains) to the southeast (e.g., the Wuyi Mountains), and from the northwest (e.g., the Dabie Mountains) to the southwest (e.g., the Luoxiao Mountains). Most of them were originally formed 200–56 million years ago (Ma) (Wan, 2012), and were dramatically reshaped by the uplift-denudation processes of the Cenozoic (Yuan et al., 2011; Shi et al., 2013; Ye et al., 2014). Previous studies have shown that the mountainous topography of East China is a key factor in determining the genetic structure of local plants (e.g., Bai et al., 2014; Wang et al., 2015; Tian et al., 2015; Zhang et al., 2016; Zhang et al., 2018). However, most studies focused on the species exhibiting a much wider distribution in subtropical China. Few of them have been conducted in detail to explore the role of those long-standing mountains in shaping the patterns of genetic variation of local plants. Overall, it remains poorly understood to which extent the interactions of persistent landscape barriers with historical climatic dynamics have influenced the genetic structure of native species in East China.

**Figure 1:**
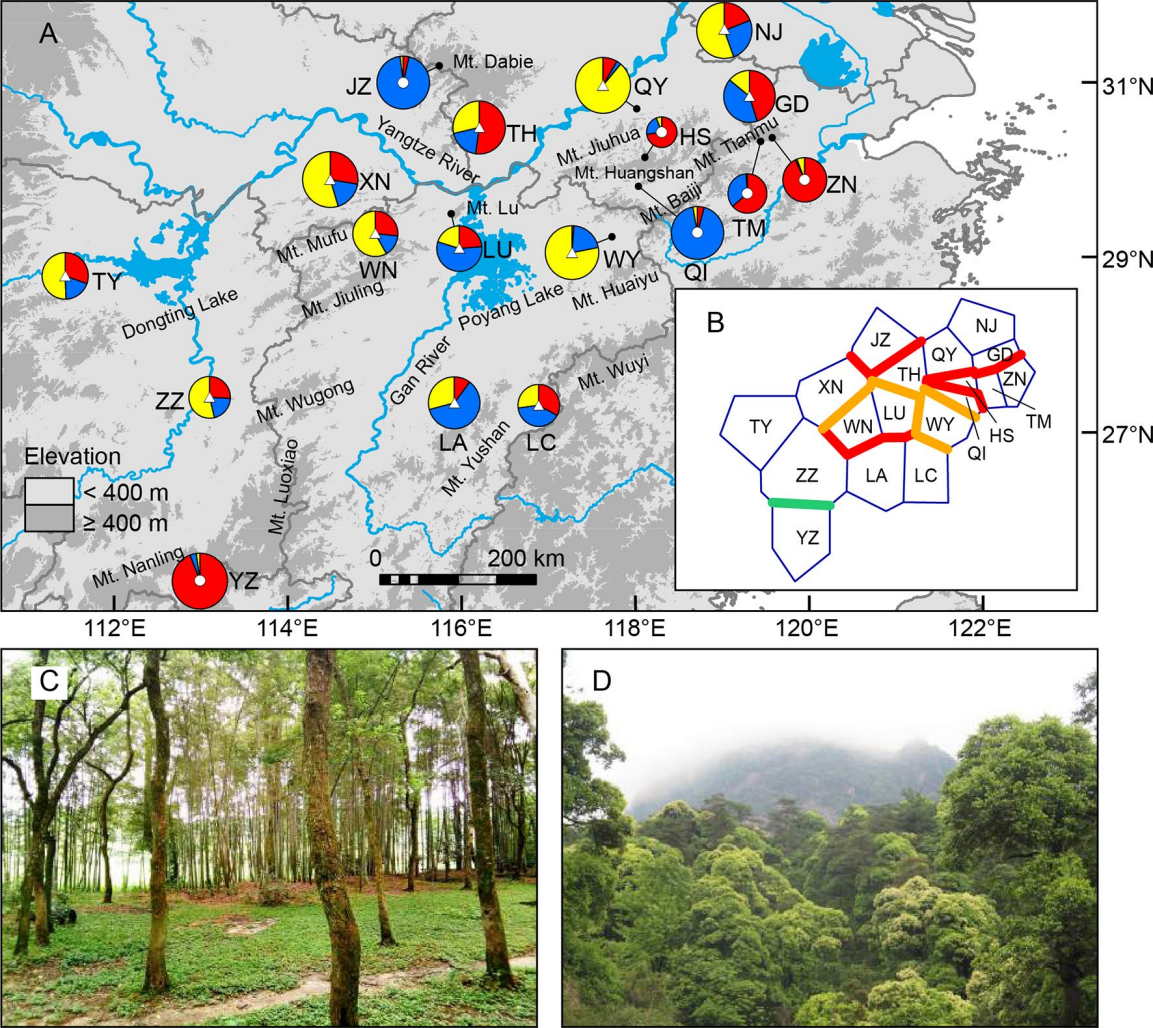
**(A)** Sampling sites of *Quercus chenii* in China, and geographical distribution of three ancestral groups corresponding to the genetic cluster I (red), II (blue), and III (yellow) as inferred by Bayesian cluster analysis based on the genetic variation at 14 nuclear microsatellite loci. Circles and triangles in the center of each pie chart indicate populations located at the high and low elevation regions, respectively. Pie chart sizes are proportional to the sample size. **(B)** Major genetic boundaries identified by BARRIER 2.2 (Manni et al., 2004). The blue lines represent the Voronoi tessellation. The red, orange, and green lines indicate geographic barriers with bootstrap support of >90%, 70%–90%, and 50%–69.9%, respectively. **(C)** and **(D)** show natural habitats of populations WN (lowlands) and HS (highlands), respectively. Population codes are shown in **Table 1** and **Supplementary Table S1**.

*Quercus chenii* Nakai is a deciduous oak species representative of the East China flora (Wu, 1979). The natural habitats of the species span numerous plains and basins, and extend over most mountain ranges mentioned above, varying from pure deciduous forests at relatively low altitudes (e.g., the Poyang Lake Basin; **Figure 1C**) to mixed evergreen and deciduous broad-leaved forests at relatively high altitudes (e.g., the Huangshan Mountains; **Figure 1D**). Furthermore, *Q. chenii* is closely related to another two oak species, *Q. acutissima* and *Q. variabilis*. They constitute the East Asian clade of section *Cerris* (Simeone et al., 2018). The first occurrence of reliable fruit fossils of the section in China came from the middle Miocene formation in Shanwang, Shandong Province (Song et al., 2000), implying that *Q. chenii* and its East Asian siblings probably have experienced a long and complicated evolutionary history driven by the geological and climatic dynamics since the Neogene. Thus, *Q. chenii* may provide us with a useful model to detect how landscape features and climatic forces have interacted over time and space to affect the patterns of genetic variation for extant plants in East China.

Here, we combine information from two sets of molecular markers, i.e., bi-parentally inherited nuclear microsatellites (nSSRs) and maternally inherited chloroplast (cp) DNA sequences, and use an integrative approach including landscape genetic, phylogeographic, fossil-calibrated phylogenetic, and ecological niche model (ENM) analyses to clarify the associations between topography, climate, and the genetic variation of *Q. chenii*. Specifically, we first analysed the genetic boundaries of nuclear variation and the geograhic patterns of cpDNA haplotypes to test whether long-standing mountains have facilitated population differentiation through geographic isolation. Second, we performed redundancy analysis (RDA) to distinguish between geographic and climatic effects on genetic divergence, and to test whether environmental isolation has also contributed to the local patterns of intraspecific variation. Third, we used approximate Bayesian computation (ABC) to explore the past demographic history, and used least-cost path calculations to infer the potential migration routes, to test whether lowlands have increased the genetic connectivity among populations especially when the species experienced range expansions. Finally, we reconstructed a fossil-calibrated phylogeny to determine the divergence times of major intraspecific lineages, and to detect how palaeoclimatic changes since the Neogene have influenced the evolutionary history of *Q. chenii*.

## Materials and Methods

### Sampling

Between May 2014 and September 2017, we collected fresh leaf material of 419 individuals from 18 populations throughout the entire distribution of *Q. chenii* in China (**Figure 1A** and **Supplementary Table S1**). In each population, eight to ten fresh leaves per tree were sampled for nine to 30 adult individuals at least 30 m apart from each other. Two species from *Quercus* section *Quercus* (*Q. fabri* and *Q. aliena*) and two species from genus *Castanea* (*C. mollissima* and *C. henryi*) were used as outgroups. Leaf tissues were quickly dried with silica gel and stored at room temperature in the laboratory. Spatially explicit information was recorded for each population using a handheld GPS unit (Magellan, USA). Voucher specimens of all individuals of *Q. chenii* and outgroups are deposited in the Herbarium of Nanjing Forestry University (HNFU) (**Supplementary Table S1**).

### DNA Extraction, Sequencing, and Microsatellite Genotyping

Total genomic DNA was extracted from 30 mg leaf tissue of each individual using a Plant Genomic DNA Kit (Tiangen, Beijing, China). Four chloroplast intergenic spacers, *atp*B-*rbc*L, *psb*A-*trn*H, *trn*S(GCU)-*trn*G(UCC), and *trn*S(GCU)-*trn*T(GGU), were sequenced for six to ten individuals per population following protocols in Zhang et al. (2015). For primer details, please refer to **Supplementary Table S2**.

All the 419 samples were genotyped at 14 nSSR loci, including quru-GA-0M07, quru-GA-1H14, quru-GA-1M17, and quru-GA-2G07 (Aldrich et al., 2002), ssrQrZAG4, ssrQrZAG7, ssrQrZAG11, ssrQrZAG59, ssrQrZAG96, and ssrQrZAG112 (Kampfer et al., 1998), ssrQpZAG15 and ssrQpZAG110 (Steinkellner et al., 1997), QM57-3M and QM67-3M1 (Isagi and Suhandono, 1997) (**Supplementary Table S3**). The forward primers of each locus were labeled with fluorescent dyes 6-FAM or HEX (Applied Biosystems, USA). PCR reactions were performed following Zhang et al. (2018), with primer-specific temperature described in **Supplementary Table S3**. PCR products were separated on an ABI3730xl automated Genetic Analyzer using ROX-500 as an internal standard (Applied Biosystems, USA). Allele sizes were determined manually using GENEMARKER 2.2.0 (Applied Biosystems, USA).

### Nuclear Microsatellite Data Analysis

#### Null Alleles, Genetic Diversity and Differentiation

The frequency of null alleles was estimated for each locus in each population using INEST 2.2 (Chybicki and Burczyk, 2009) based on the full model, which accounts for null alleles (*n*), inbreeding (*f*), and genotyping failures (*b*) simultaneously. The statistical significance was tested by comparing deviance information criterion (DIC) values between the full model and the null allele-ignored model (*n* is fixed at 0). Linkage disequilibrium (LD) for all locus pairs and deviation from Hardy-Weinberg equilibrium (HWE) in each population were tested by randomization in FSTAT 2.9.3.2 (Goudet, 2001), with *P*-values adjusted by Bonferroni correction (Rice, 1989). Genetic diversity statistics such as allelic richness (*A*_R_) and genetic diversity within populations (*H*_S_) were estimated for each population, and compared between highland and lowland groups (**Table 1**) that were defined by the results of STRUCTURE (see below). The two-sided *P*-values were obtained after 10,000 permutations.

**Table 1:** Genetic statistics for 18 populations of *Quercus chenii* based on the genetic variation of chloroplast (cp) DNA sequences and nuclear microsatellite (nSSR) markers.

P	E (m)	nSSR						cpDNA		
		n_SSR_	Null	A_R_	H_S_	F_IS_	A_D_	n*_cp_*	H*_d_*	Haplotypes
**Highland populations**										
YZ	247	30	0.00	4.394	0.634	−0.078	0.005	10	0	H8(10)
HS	450	9	0.02	4.000	0.637	0.054	0.339	9	0	H18(9)
TM	459	15	0.02^†^	3.849	0.585	0.015	0.410	10	0	H18(10)
QI	494	27	0.00	4.464	0.608	−0.093	0.023	10	0	H9(10)
ZN	496	19	0.01	4.733	0.617	0.056	0.025	10	0	H14(10)
JZ	625	27	0.01^†^	4.210	0.609	−0.104	0.000	10	0	H1(10)
**Lowland populations**										
XN	37	30	0.01	5.718	0.710	−0.006	0.640	10	0	H5(10)
GD	50	26	0.01	5.509	0.673	−0.065	0.683	10	0	H1(10)
QY	56	30	0.03^†^	5.901	0.713	0.068	0.089	10	0	H15(10)
WN	82	20	0.01	5.489	0.680	0.023	0.580	10	0	H12(10)
ZZ	84	17	0.02^†^	5.705	0.710	0.036	0.661	6	0	H1(6)
WY	91	28	0.02^†^	5.890	0.685	0.043	0.245	10	0.533	H6(6), H7(4)
LU	99	20	0.01	5.323	0.659	0.002	0.625	10	0.200	H6(9), H13(1)
TH	99	27	0.02^†^	5.432	0.700	−0.021	0.679	10	0	H10(10)
LC	102	17	0.01	5.354	0.699	0.002	0.879	10	0.200	H1(1), H11(9)
TY	139	21	0.01	5.266	0.686	0.014	0.698	10	0.711	H1(5), H2(1), H3(3), H4(1)
NJ	149	30	0.02	5.898	0.708	0.113*	0.632	10	0.622	H1(6), H16(2). H17(2)
LA	175	26	0.01^†^	4.785	0.661	−0.043	0.520	10	0	H1(10)

P, population code; E, elevation; n_SSR_, sample sizes for nuclear microsatellite markers; Null, null allele frequency averaged across the 14 nSSR loci,† indicates the significance of null alleles in the full model, which accounts for null alleles (n), inbreeding (f) and genotyping failures (b) simultaneously; A_R_, allelic richness with rarefaction to the common sample size of 9; H_S_, genetic diversity within populations averaged across loci; F_IS_, inbreeding coefficient, * indicates P < 0.05 after Bonferroni correction; A_D_, genetic admixture index; n_cp_, sample sizes for chloroplast DNA sequences; H_d_, haplotype diversity.

Genetic differentiation between each pair of populations was determined by *F*_ST_ (Weir and Cockerham, 1984) using MSA 4.05 (Dieringer and Schlötterer, 2003), and was visually displayed with the *heatmap.2* function in R package ‘gplots’ (Warnes et al., 2016). Global *F*_ST_ and the standardized measure, *G*′_ST_ (Hedrick, 2005) were calculated for each locus and over all loci using MSA. The statistical significance was tested by 10,000 permutations. We also performed analysis of molecular variance (AMOVA) to estimate the genetic differentiation between highland and lowland populations, with the significance of fixation indices (*F*_CT,_ *F*_SC_, and *F*_ST_) tested by 10,000 permutations in ARLEQUIN 3.5 (Excoffier and Lischer, 2010).

#### Genetic Structure and Demographic History

Following recommendations of Hubisz et al. (2009), an admixture model with sampling locations as prior was implemented in STRUCTURE 2.3.4 (Pritchard et al., 2000) to detect the underlying genetic structure of *Q. chenii*. Twenty independent runs were performed for each number of clusters (*K*) from 1 to 11 with 500,000 iterations as burn-in followed by 500,000 Markov Chain Monte Carlo (MCMC) iterations. Both the Ln Pr(*X*|*K*) selection method (Pritchard et al., 2000) and the Δ*K* method (Evanno et al., 2005) as implemented in STRUCTURE HARVESTER (Earl and vonHoldt, 2012) were used to determine the optimal number of clusters (Gilbert et al., 2012; Janes et al., 2017). Individual membership coefficients were post-processed and visually displayed for each value of *K*, and for both major and minor modes within 20 total runs of a given *K* (Janes et al., 2017) with the CLUMPAK web server (Kopelman et al., 2015). We also employed Monmonier’s maximum-difference algorithm in BARRIER 2.2 (Manni et al., 2004) to delineate potential biogeographic boundaries that may shape the genetic structure of the species across landscapes. Robustness of barriers was estimated by applying the algorithm to 10,000 Nei’s distance matrices obtained through bootstrapping over all loci with MSA 4.05.

**Figure 2:**
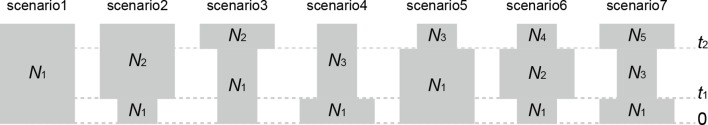
Seven demographic scenarios compared in approximate Bayesian computation (ABC) for both highland and lowland populations of *Quercus chenii*. (1) constant effective population size at both *t*_1_ and *t*_2_; (2) a recent bottleneck at *t*_1_; (3) an old bottleneck at *t*_2_; (4) a recent expansion at *t*_1_; (5) an old expansion at *t*_2_; (6) an old expansion at *t*_2_ followed by a recent bottleneck at *t*_1_; and (7) an old bottleneck at *t*_2_ followed by a recent expansion at *t*_1_. Parameter abbreviations include effective population sizes (*N*_1_–*N*_5_) and generation-scaled times (*t*_1_ and *t_2_*).

To explore the past demographic history of *Q. chenii*, seven competing evolutionary scenarios (**Figure 2**) were compared for both highland and lowland populations using approximate Bayesian computation (ABC) as implemented in DIYABC 2.1.0 (Cornuet et al., 2014). One million simulations were run for each scenario. Four summary statistics and prior distributions of seven historical parameters are shown in **Supplementary Tables S4** and **S5**. Model checking process was performed through principal component analysis (PCA). The 1% simulated data closest to the observed data was used to estimate the posterior distributions of historical parameters and the relative posterior probabilities of each scenario *via* a logistic regression approach. An average generation time of 100 years was used to estimate the times of evolutionary events (Cavender-Bares et al., 2011; Zeng et al., 2015). To assess the effect of probable reduction in population size on genetic diversity due to bottlenecks, heterozygosity excess was tested for each population under two mutation models, the stepwise mutation model (SMM), and the two-phase mutation model (TPM) with 70% stepwise mutations and 30% multistep mutations. The statistical significance was obtained by Wilcoxon sign-rank test with 10,000 simulations in BOTTLENECK 1.2.02 (Cornuet and Luikart, 1996).

#### Associations of Genetic Statistics With Geography and Climate

We analyzed relationships of genetic diversity statistics (*A*_R_ and *H*_S_) and genetic admixture index (*A*_D_) with the geographical locations (latitude and longitude) and climatic conditions of each population using a general linear model (GLM). Genetic admixture index was calculated as the standard deviation (SD) of probabilities of population membership (*Q*) to each of the three genetic clusters (*K* = 3) inferred by STRUCTURE, and normalized to a range of 0 to 1 following the method of Ortego et al. (2015). Eight climatic predictors that show low to moderate correlations (| *r* | < 0.70; **Supplementary Table S6**) were selected and extracted for each population from the Worldclim database (http://www.worldclim.org/) at 30″ resolution for the present. Elevation was also used as a climatic variable because it captures vertical variation of microclimatic features (Wanderley et al., 2018). All explanatory variables were centered and scaled to have zero mean and unit variance. GLMs with a normal error structure and an identity link function were applied to three predictor matrices: (1) latitude and longitude; (2) elevation and eight climatic variables; (3) only eight climatic variables. For each dataset, the best combination of variables that describe the relationship with genetic statistics was selected by a backward elimination algorithm based on Akaike information criterion (AIC) scores. Sample size was taken into account using a weighted least square method. Variance inflation factors (VIFs) were used to quantify the severity of multicollinearity in each model. All statistical analyses were conducted in R 3.5.1 (R Core Team, 2018).

#### Effects of Climate and Geography on Pattern of Genetic Variation

To test the role of geography and climate in shaping the patterns of present genetic variation, we performed redundancy analysis (RDA) and partial RDA using R package ‘vegan’ (Oksanen et al., 2018). RDA provides a powerful tool for detecting multivariate genotype-environment associations and shows better performance than widely used methods like Mantel test (Legendre and Fortin, 2010; Forester et al., 2018). A total of 177 response variables were generated by converting the microsatellite data into allele frequencies, with one allele removed from each locus (Smouse and Williams, 1982). Obtained allelic variables were Hellinger-transformed (Legendre and Gallagher, 2001) to be suitable to fit RDA models using the *decostand* function in R package ‘vegan’. Eigenvectors corresponding to positive eigenvalues of the principal coordinates of neighbor matrix (PCNM) were used as geographic variables to quantify the spatial pattern in a rectangular form (Borcard and Legendre, 2002). PCNM was obtained from a truncated matrix of great circle distances between sampled individuals *via* the *pcnm* function in R package ‘vegan’. Pairwise great circle distances were calculated with the *distVincentyEllipsoid* function in R package ‘geosphere’ (Hijmans, 2017). An equal number of geographic and climatic variables were selected to avoid bias toward the effects of isolation by distance (IBD) or isolation by environment (IBE). A limit of the total number less than the number of populations was used to reduce over-parameterization (Wanderley et al., 2018). Based on these criteria, variables were dropped off when exhibiting lowest values of adjusted coefficient of determination (*R*_adj_^2^) in the forward stepwise selection for partial RDA models including all the geographic variables (PCNM1–PCNM10) or climatic variables (**Supplementary Table S6**). Finally, a total of 16 predictors (**Supplementary Table S7**) were applied to the full RDA model. Partial RDA of geographic variables with climatic effect controlled, and partial RDA of climatic variables with geographic effect controlled, were conducted to determine the proportions of genetic variance explained by geographic and climatic variables. Model significance was tested by 999 permutations.

### Chloroplast DNA Sequence Analysis

#### Genetic Diversity, Differentiation and Phylogeographic Structure

Sequences of the four cpDNA fragments were proofread and aligned in BIOEDIT 7.2.5 (Hall, 1999), and then concatenated into a single locus for each sample. Indels were coded as binary characters (0/1) according to the simple gap coding method (Simmons and Ochoterena, 2000) as implemented in GAPCODER (Young and Healy, 2003). Chloroplast haplotypes were determined by DNASP 5.10 (Librado and Rozas, 2009), and a median-joining network was then inferred using POPART 1.7 (Leigh and Bryant, 2015). Genetic diversity statistics were estimated for the whole species (average genetic diversity within populations, *h*_S_; total genetic diversity, *h*_T_) with PERMUT 2.0 (Pons and Petit, 1996), and for each population (haplotype diversity, *H*_d_) with DNASP 5.10. Genetic differentiation between highland and lowland populations was evaluated by AMOVA using ARLEQUIN 3.5. The phylogeographic structure was assessed by comparing the difference between two measures of population differentiation (*G*_ST_ and *N*_ST_) with 10,000 permutations in PERMUT 2.0. Mismatch distribution analysis (under the demographic expansion model), Tajima’s *D* and Fu’s *F*_S_ test were performed through ARLEQUIN 3.5 to detect the demographic expansion for the species.

#### Phylogenetic Relationship and Divergence Time

Phylogenetic relationships and divergence times among cpDNA haplotype lineages were estimated using Bayesian inference (BI) as implemented in BEAST 1.8.4 (Drummond and Rambaut, 2007). We applied a GTR + I substitution model selected by JMODELTEST 2.1.10 (Darriba et al., 2012) to the nucleotide partition, and a stochastic Dollo model to the binary coded indel partition. Two calibration points were chosen based on fossil records and recent phylogenetic studies of oaks. First, the earliest unequivocal megafossil of subfamily Castaneoideae of Fagaceae from the Paleocene/Eocene boundary (Crepet and Nixon,1989) was used to set the root age to 53.50 Ma (normal prior, SD = 3 Ma) for all members of *Quercus* and *Castanea*. Second, we used the result from the most recent phylogenetic study of East Asian oaks (Deng et al., 2018) as a secondary calibration point to constrain the crown age of *Quercus* (including section *Cerris* and section *Quercus*) to 35.89 Ma (normal prior, SD = 2 Ma). A combination of uncorrelated lognormal relaxed clock and Bayes-skyline coalescent prior was selected for node age estimation through Bayes factor (BF) calculations based on marginal likelihoods obtained by stepping-stone sampling and path sampling. For the preferred combination, two independent MCMC runs were performed, each with 100 million generations and sampled every 10,000 generations. Tree and log files were combined through LOGCOMBINER 1.8.4, and then passed to TRACER 1.7.1 (Rambaut et al., 2018) for assessing the convergence, and to TREEANNOTATOR 1.8.4 for constructing a maximum clade credibility tree with a posterior probability limit of 0.5 and the first 25% generations discarded as burn-in.

### Ecological Niche Modeling and Dispersal Corridors

We employed a maximum entropy approach in MAXENT 3.4.1 (Phillips et al., 2018) to simulate the modern distribution of *Q. chenii*. Species occurrence records were mainly collected from the fieldwork, the literature, and the database of the Global Biodiversity Information Facility (GBIF, https://www.gbif.org/), the Chinese Virtual Herbarium (CVH, http://www.cvh.ac.cn/) and the Plant Photo Bank of China (PPBC, http://www.plantphoto.cn/). We filtered the data by removing duplicate records and retaining only one record among all locations falling within the same 2.5′ × 2.5′ grid. Finally, a total of 54 occurrence points were obtained for *Q. chenii*. The environmental layers of the eight bioclimatic variables (**Supplementary Table S6**) were downloaded from the Worldclim database (http://www.worldclim.org/) with a resolution of 30″ for the present, the Mid Holocene (∼6 ka), the Last Glacial Maximum (LGM, ∼22 ka), and the Last Interglacial (LIG, ∼120–140 ka) under the Community Climate System Model 4 (CCSM4). All layers were clipped to the same spatial range (15°–40° N, 105°–140° E). The optimal settings of feature types (linear and quadratic) and regularization multiplier (value = 1) were selected through the R package ‘ENMeval’ (Muscarella et al., 2014). Model performance was evaluated using the areas under the receiver operating characteristic curve (AUC) produced by 10-fold cross-validation. The generated models were then projected onto the other three scenarios of past climates. Obtained layers were used to calculate the average, minimum, and 1-SD of occurrence probabilities across different historical periods in ARCMAP 10.1 (ESRI). The resulting layers were reclassified to show putative areas with moderate and high ecological stability (Chan et al., 2011), using 80% and 90% (for average), and 50% and 80% (for minimum and 1-SD) of the maximum values as thresholds. To assess the landscape connectivity of the cpDNA haplotype network, we applied SDMTOOLBOX (Brown, 2014) in ARCMAP 10.1 (ESRI) to convert species distribution models to ‘dispersal cost’ layers, and then to create a raster of the sum of least-cost corridors between populations that share haplotypes.

## Results

### Nuclear Microsatellite Diversity, Differentiation, Genetic Structure, and Demographic History

Using INEST, the frequency of null alleles was estimated to be lower than the threshold of 0.05 at each of the 14 loci across populations (**Table 1**). Only three of the 91 locus pairs showed significant LD in two populations (quru-GA-1M17 × ssrQrZAG4 in QI, ssrQrZAG59 × ssrQrZAG4 in QI, and quru-GA-1M17 × ssrQpZAG15 in TH; *P* < 0.05 after Bonferroni correction). No consistent genotypic disequilibrium was found between any locus pairs across all populations, so all loci were used for further analyses. Significant deviation from HWE was only detected in one of the 18 populations (*P* < 0.05 after Bonferroni correction; **Table 1**).

**Figure 3:**
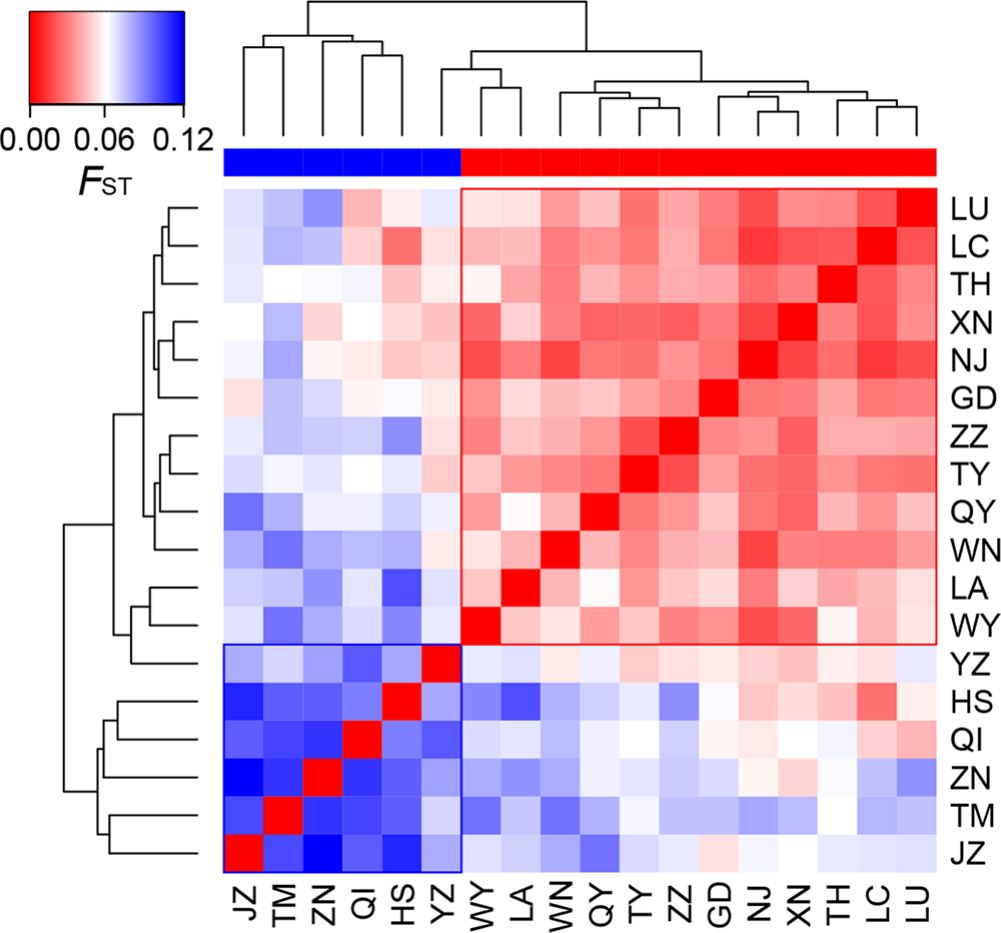
Heatmap of pairwise *F*_ST_ values among the 18 populations of *Quercus chenii*. Blue and red bars indicate highland and lowland populations, respectively. Population codes are shown in **Table 1** and **Supplementary Table S1**.

At the population level, allelic richness (*A*_R_) ranged from 3.849 to 5.901, and genetic diversity within populations (*H*_S_) varied from 0.585 to 0.713 (**Table 1**). Highland populations presented lower genetic diversity than lowland populations (*P* < 0.001 for both *A*_R_ and *H*_S_; **Table 2**). Genetic differentiation among populations was significant over all loci (*F*_ST_ = 0.054, *P* < 0.001; *G*′_ST_ = 0.228, *P* < 0.001; **Supplementary Table S3**). Pairwise *F*_ST_ ranged from 0.015 to 0.124, with 152 of all the 153 pairs being significant (*P* < 0.05 after Bonferroni correction). The highest pairwise *F*_ST_ values were observed for JZ-ZN, corresponding to two montane habitats located at the northwest (the Dabie Mountains) and southeast (the Tianmu Mountains). A higher level of genetic differentiation was detected among highland populations (*F*_ST_ = 0.099, range of pairwise *F*_ST_: 0.072–0.124) than among lowland populations (*F*_ST_ = 0.036; range of pairwise *F*_ST_: 0.015–0.059) (**Table 2** and **Figure 3**). Genetic differentiation between these two groups was low but significant (*F*_CT_ = 0.004, *P* = 0.013), with only 0.43% of the variation partitioned among groups, and the most (94.33%) partitioned within populations (**Table 3**).

**Table 2:** Comparison of allelic richness (*A*_R_), genetic diversity within populations (*H*_S_), genetic differentiation among populations (*F*_ST_), and genetic admixture index (*A*_D_) between highland and lowland populations of *Quercus chenii*.

Group	A_R_	H_S_	F_ST_	A_D_
Highlands	4.275	0.615	0.099	0.134
Lowlands	5.522	0.691	0.036	0.578
*P*-value	0.000	0.000	0.000	0.001

Two-sided P-values for A_R_, H_S_, and F_ST_ were obtained after 10,000 permutations using FSTAT. The P-value for A_D_ was obtained through t-test.

**Table 3:** Analyses of molecular variance (AMOVAs) based on chloroplast (cp) DNA haplotype frequencies and nuclear microsatellite (nSSR) allele frequencies for populations of *Quercus chenii*.

Source of variation	cpDNA					nSSR				
	df	SS	VC	Variation (%)	Fixation index	df	SS	VC	Variation (%)	Fixation index
***All populations***										
Among populations	17	348.19	2.08	88.65		17	291.67	0.27	5.44	
Within populations	157	41.80	0.27	11.35	*F*_ST_ = 0.887**	820	3835.28	4.68	94.56	*F*_ST_ = 0.054**
***Highlands and lowlands***										
Among groups	1	21.76	0.01	0.60	*F*_CT_ = 0.006	1	24.84	0.02	0.43	*F*_CT_ = 0.004*
Among populations within groups	16	326.43	2.07	88.09	*F*_SC_ = 0.886**	16	266.83	0.26	5.23	*F*_SC_ = 0.053**
Within populations	157	41.80	0.27	11.31	*F*_ST_ = 0.887**	820	3835.28	4.68	94.33	*F*_ST_ = 0.057**

df, degree of freedom; SS, sum of squares; VC, variance components; **P < 0.01. *P < 0.05. P-value was obtained through 10,000 permutations in ARLEQUIN.

Bayesian cluster analysis showed that the highest Δ*K* occurred at *K* = 2 and 3 (**Supplementary Figure S1**). When *K* = 3, a significant decline in the probability of membership to genetic cluster III (*Q*_III_) in each population was detected with increasing elevation (*R* = 0.711, *P* = 0.001) (**Supplementary Figure S2**). According to this trend, we divided all populations into two groups: (1) Highland populations. Cluster III presented a much lower *Q* value ranging from 0.01 to 0.06. Four of those were dominated by genetic cluster I (*Q*_I_: 0.64–0.94), and two were dominated by cluster II (*Q*_II_: 0.93–0.94) (**Figure 1A**, **Supplementary Table S8** and **Figure S3**). (2) Lowland populations. Cluster III presented a higher *Q* value varying from 0.14 to 0.89. The other two clusters showed a *Q* value lower than or equal to 0.60 (**Figure 1A**, **Supplementary Table S8** and **Figure S3**). A higher level of genetic admixture was detected in lowland populations (mean of *A*_D_ = 0.578) than in highland populations (mean of *A*_D_ = 0.134) (**Table 2**). The BARRIER analysis revealed multiple genetic boundaries that were well associated with persistent landscape barriers of East China. Among those, highland populations were isolated from lowland ones by the Dabie Mountains, the Jiuhua Mountains, the Baiji Mountains, and the Tianmu Mountains, with more than 70% bootstrap support. Lowland populations inside the Huaiyu Mountains, the Mufu Mountains, the Jiuling Mountains, and the Lu Mountains were also separated from the adjacent ones outside with more than 70% bootstrap support (**Figure 1B**).

ABC analyses clearly favoured the hypothesis that *Q. chenii* may have experienced a recent demographic expansion (scenario 4) within both high and low elevation regions (**Supplementary Table S9**). All the summary statistics together with PCA plots indicated a good fit of this scenario to the observed data (**Supplementary Table S4** and **Figure S4**). Assuming an average generation time of 100 years, the recent expansion event was dated to 0.121 Ma (95% CI: 0.008–0.290 Ma) for the highland populations, with effective population size varying from 17,600 (95% CI: 868–243,000) to 526,000 (95% CI: 41,800–977,000). For the lowland populations, the recent expansion event was dated to 0.138 Ma (95% CI: 0.010–0.291 Ma), with effective population size varying from 12,800 (95% CI: 604–196,000) to 533,000 (95% CI: 46,000–977,000) (**Table 4** and **Supplementary Figure S5**). Both times corresponded to the Last Interglacial (∼0.14–0.12 Ma). Significant evidence of recent bottleneck was only detected in populations HS and TH under the two-phase mutation model (TPM) (*P* < 0.05; **Supplementary Table S8**).

**Table 4:** Posterior distributions of historical parameters for scenario 4 in ABC analyses.

Parameter	Highland populations				Lowland populations			
	median	mean	95% low	95% high	median	mean	95% low	95% high
*N*_1_	526,000	519,000	41,800	977,000	533,000	525,000	46,000	977,000
*N*_3_	17,600	41,400	868	243,000	12,800	31,800	604	196,000
*t*_1_	0.121	0.132	0.078	0.290	0.138	0.143	0.096	0.291

N_1_, current effective population size; N_3_, effective population size before the expansion event; t_1_, time of the expansion event (Ma).

### Associations of Genetic Statistics With Geography and Climate

**Figure 4:**
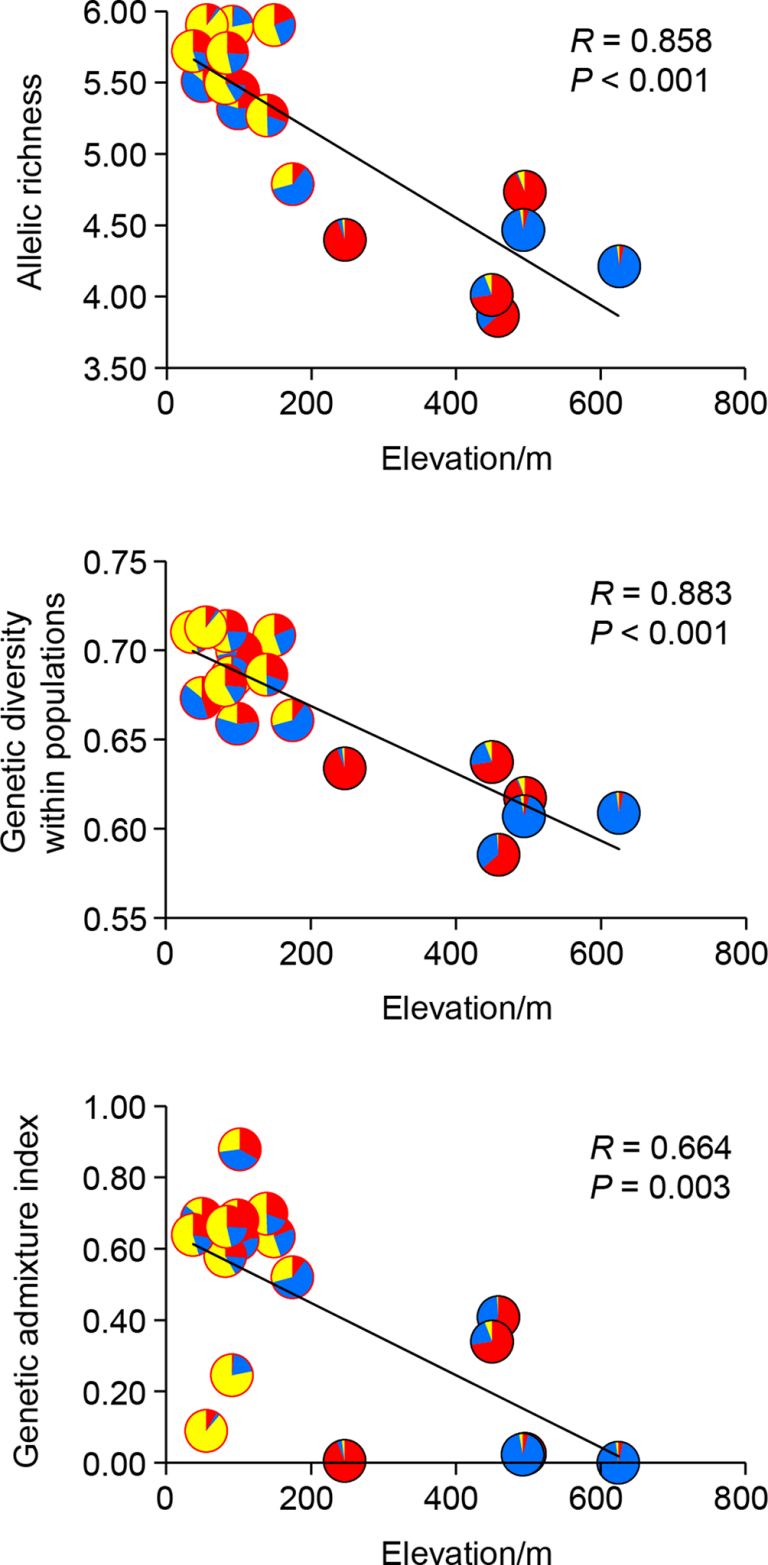
Linear correlations between allelic richness (*A*_R_), genetic diversity within populations (*H*_S_), genetic admixture index (*A*_D_) and elevation of each population of *Quercus chenii*. Pie charts show proportions of the genetic cluster I (red), II (blue), and III (yellow) as inferred by Bayesian cluster analysis for each population. Red and black circles indicate lowland and highland populations, respectively.

Genetic diversity statistics (*A*_R_ and *H*_S_) and genetic admixture index (*A*_D_) were not associated with longitude or latitude (all *P*-values > 0.05), but correlated with elevation and climatic variables. The model that best explained genetic diversity contained elevation (all *P*-values < 0.01), with mean diurnal temperature range (bio2), temperature seasonality (bio4), and mean temperature of driest quarter (bio9) as covariates. The model that best explained *A*_D_ included only elevation (*P* < 0.01) (**Table 5**). Significant negative correlations between elevation and these three genetic statistics were also revealed by simple linear regression (all *P*-values < 0.01, **Figure 4**). When using pure climatic predictors to describe these relationships, annual mean temperature (bio1) and temperature seasonality (bio4) remained in all the best-fit models. Significant positive associations of bio1 and bio4 were detected with *A*_R_, *H*_S_, and *A*_D_ (all *P*-values < 0.05). Other covariates, including mean diurnal temperature range (bio2) and mean temperature of wettest quarter (bio8), showed marginally significant (0.05 < *P* < 0.10) or insignificant (*P* > 0.10) positive correlations with genetic diversity (**Table 5**). Multicollinearity was not detected in all the models as indicated by VIF < 2 for each variable (**Table 5**).

**Table 5:** General linear models (GLMs) showing relationships of allelic richness (*A*_R_), genetic diversity within populations (*H*_S_), and genetic admixture index (*A*_D_) with elevation and climate for each population of *Quercus chenii*. The best combination of variables was selected by a backward elimination algorithm based on Akaike information criterion (AIC) scores.

	Estimate	SE	*t*	*P*	VIF	AIC
	**Model: *H*_S_ ∼ climate + elevation**					
(intercept)	0.665	0.004	156.317	0.000***	–	−88.091
elevation	−0.038	0.005	−8.418	0.000***	1.061	
bio9	−0.010	0.005	−2.025	0.061*	1.061	
	**Model: *H*_S_ ∼ climate**					
(Intercept)	0.664	0.005	120.850	0.000***	–	−78.337
bio1	0.034	0.007	4.861	0.000***	1.438	
bio2	0.009	0.006	1.367	0.193	1.112	
bio4	0.033	0.006	5.365	0.000***	1.393	
	**Model: *A*_R_ ∼ climate + elevation**					
(Intercept)	5.122	0.062	82.456	0.000***	–	9.001
elevation	−0.473	0.069	−6.889	0.000***	1.142	
bio2	0.133	0.075	1.781	0.097*	1.158	
bio4	0.260	0.063	4.106	0.001***	1.160	
	**Model: *A*_R_ ∼ climate**					
(Intercept)	5.102	0.077	66.068	0.000***	–	17.252
bio1	0.484	0.097	4.979	0.000***	1.444	
bio2	0.194	0.091	2.143	0.052*	1.121	
bio4	0.558	0.091	6.113	0.000***	1.575	
bio8	0.112	0.089	1.255	0.231	1.176	
	**Model: *A*_D_ ∼ climate + elevation**					
(Intercept)	0.398	0.055	7.191	0.000***	–	3.504
elevation	−0.198	0.058	−3.432	0.003***	–	
	**Model: *A*_D_ ∼ climate**					
(Intercept)	0.398	0.063	6.280	0.000***	–	9.091
bio1	0.174	0.079	2.215	0.043**	1.376	
bio4	0.154	0.071	2.177	0.046**	1.376	

SE, standard error; VIF, variance inflation factor, VIF < 2 indicates that multicollinearity does not confound the interpretation of individual predictors in the models; bio1, annual mean temperature; bio2, mean diurnal temperature range; bio4, temperature seasonality; bio8, mean temperature of wettest quarter; bio9, mean temperature of driest quarter; ***, P < 0.01; **, P < 0.05; *, P < 0.10

### Effects of Climate and Geography on Pattern of Genetic Variation

**Figure 5:**
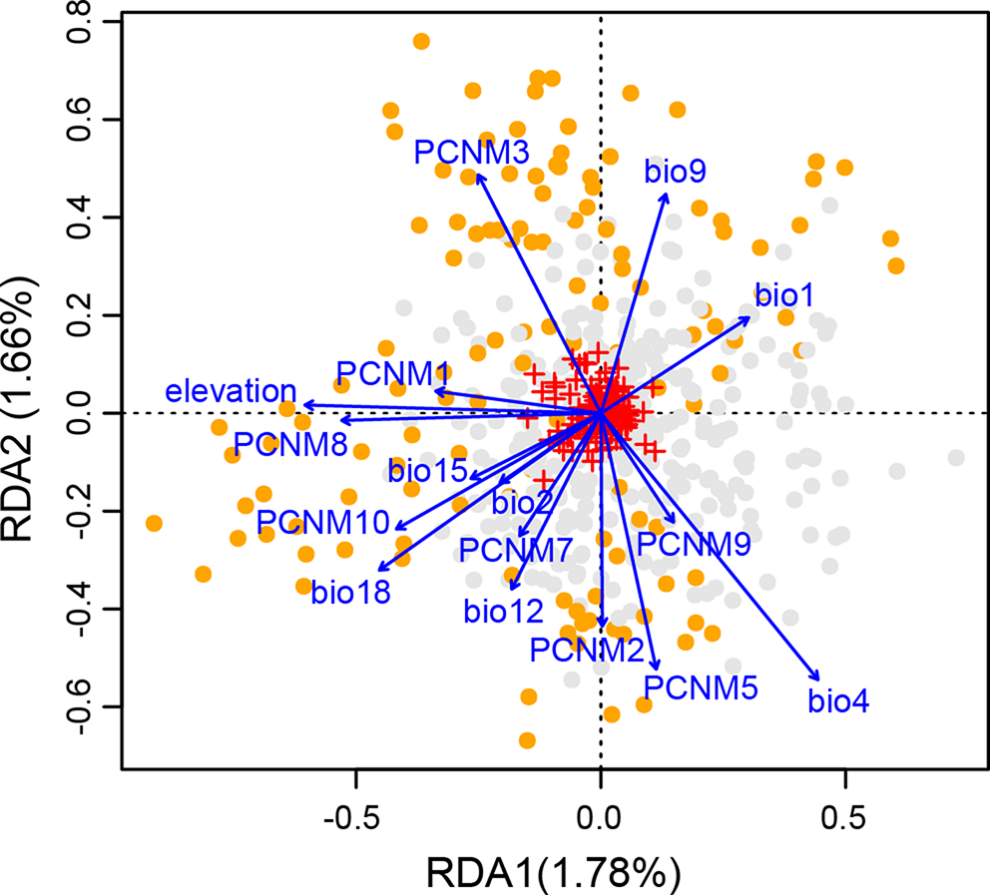
Biplot from redundancy analysis (RDA) showing the association of genetic variation at the 14 nuclear microsatellite (nSSR) loci with geographic and climatic variables. Small red crosses at the center represent the 177 allelic variables. Eight eigenvectors corresponding to positive eigenvalues of the principal coordinates of neighbor matrix (PCNM) were used as geographic variables. Eight climatic variables include elevation, annual mean temperature (bio1), mean diurnal temperature range (bio2), temperature seasonality (bio4), mean temperature of driest quarter (bio9), annual precipitation (bio12), precipitation seasonality (bio15), and precipitation of warmest quarter (bio18). Orange and gray dots indicate individuals from highlands and lowlands, respectively. The proportion of total genetic variation explained by each axis is shown in parentheses.

The full RDA model (*df* = 16, *F* = 3.726, *R*_adj_^2^ = 0.094, *P* = 0.001), and both partial RDA models corresponding to geography (*df* = 8, *F* = 3.591, *R*_adj_^2^ = 0.046, *P* = 0.001) and climate (*df* = 8, *F* = 3.518, *R*_adj_^2^ = 0.044, *P* = 0.001) were significant. The full model explained 12.91% of the total variance. The percentages of genetic variance attributed to geography and climate alone were 6.22% and 6.10%, respectively. Only 0.59% of the overall genetic variance was explained by the collinearity between geographic and climatic variables (**Supplementary Table S10**). In the full model, all the eight spatial descriptors and the eight climatic predictors were significant (all *P*-values = 0.001; **Supplementary Table S7**). Forward stepwise selection also kept all the 16 variables in the best model based on the adjusted coefficient of determination (*R*_adj_^2^). Among those, three showed the highest scores on the first three axes, i.e., elevation on the RDA1, temperature seasonality (bio4) on the RDA2, and PCNM9 on the RDA3 (**Figure 5** and **Supplementary Table S7**).

### Chloroplast DNA Diversity, Differentiation, Phylogeographic Structure, and Phylogenetic Relationship

**Figure 6:**
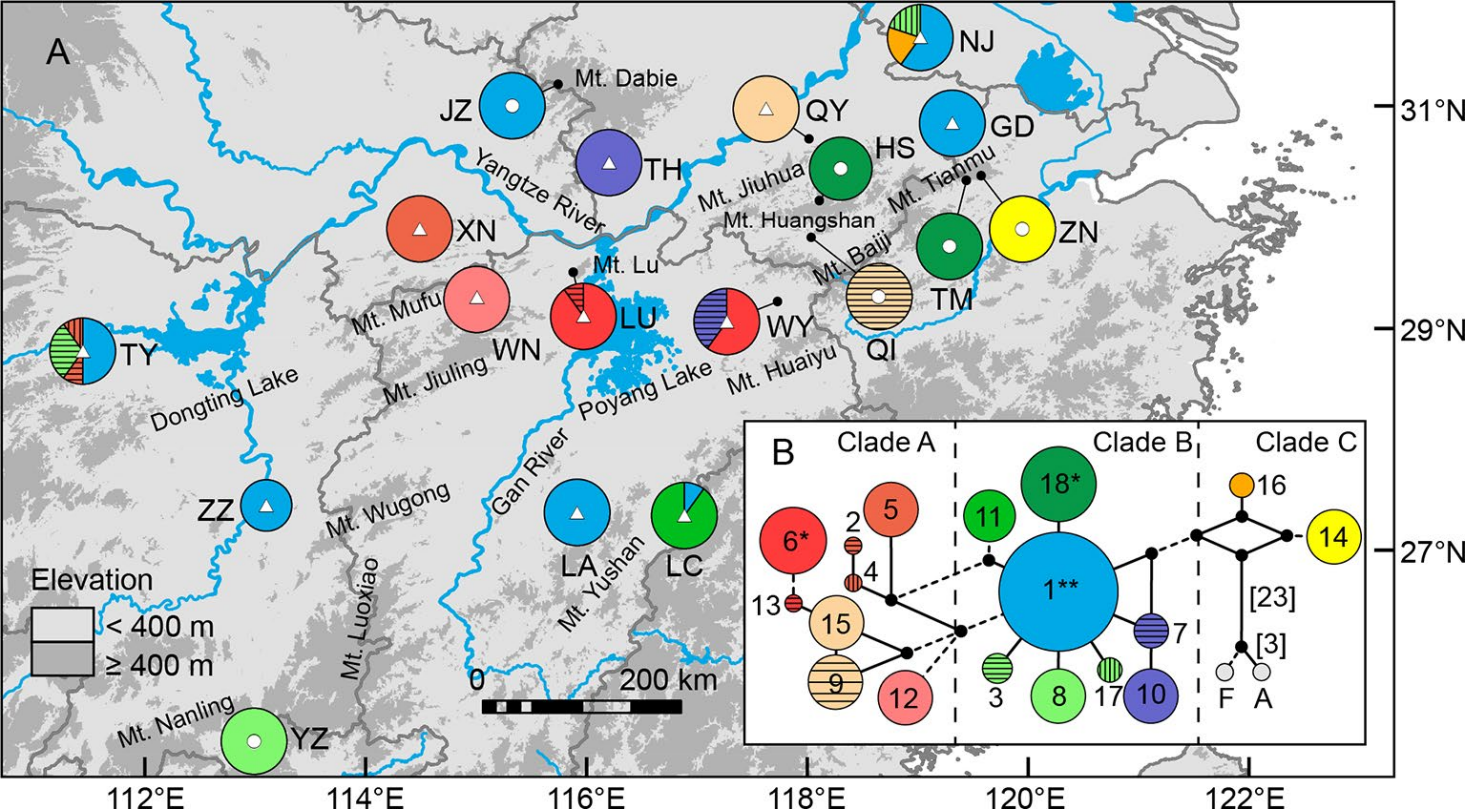
Geographical distribution **(A)** and median-joining network **(B)** of 18 chloroplast DNA haplotypes of *Quercus chenii*. Circles and triangles in the center of each pie chart in **(A)** indicate populations located at the high and low elevation regions, respectively. Population codes are shown in **Table 1** and **Supplementary Table S1**. Circle sizes in **(B)** are proportional to the frequency of a haplotype across all populations. The small black dots indicate inferred intermediate haplotypes not detected in this investigation. Dash lines indicate two mutations between haplotypes. When branches represent more than two mutations, numbers of mutations are labeled in brackets. F and A represent outgroups, *Q. fabri* and *Q. aliena*, respectively. **, shared by seven populations; *, shared by two populations.

The lengths of consensus sequences after alignment of *atp*B-*rbc*L, *psb*A-*trn*H, *trn*S(GCU)-*trn*G(UCC), *trn*S(GCU)-*trn*T(GGU), and concatenated cpDNA were 718, 639, 607, 902, and 2,866 bp, respectively (**Supplementary Table S2**). **Supplementary Table S11** Eighteen haplotypes identified in this study based on 16 nucleotide substitutions and six indels. Among those, only one (H1) was shared by seven populations, two (H6 and H18) were shared by two populations, and the other 15 were private to a single population. More than 70% populations were fixed for a single haplotype (**Figure 6**). Such a geographic pattern of haplotypes resulted in a much lower average within-population genetic diversity (*h*_S_ = 0.126) compared with the total genetic diversity (*h*_T_ = 0.917) at the species level. Intraspecific differentiation at cpDNA markers was significant (*F*_ST_ = 0.887, *P* < 0.001), with 88.65% of the total genetic variation partitioned among populations, and 11.35% partitioned within populations (**Table 3**). However, we did not detect any phylogeographic structure across the entire distribution (*N*_ST_ = 0.888 > *G*_ST_ = 0.863, *P* = 0.453). The genetic divergence between highlands and lowlands was also not significant (*F*_CT_ = 0.006, *P* > 0.05). Although the mismatch distribution for all haplotypes was bimodal**(Supplementary Figure S6**), both sum of squared deviation (0.017, *P* = 0.222) and Harpending’s Raggedness index (0.043, *P* = 0.140) did not reject the sudden expansion model. Following Qiu et al. (2009b), this statistical fit of the expansion model is here not taken as strong evidence of expansion. Tajima’s *D* test (Tajima’s *D* = −0.210, *P* = 0.475) and Fu’s *F*_S_ test (Fu’s *F*_S_ = −0.257, *P* = 0.548) also did not show evidence of extensive demographic expansion.

**Figure 7:**
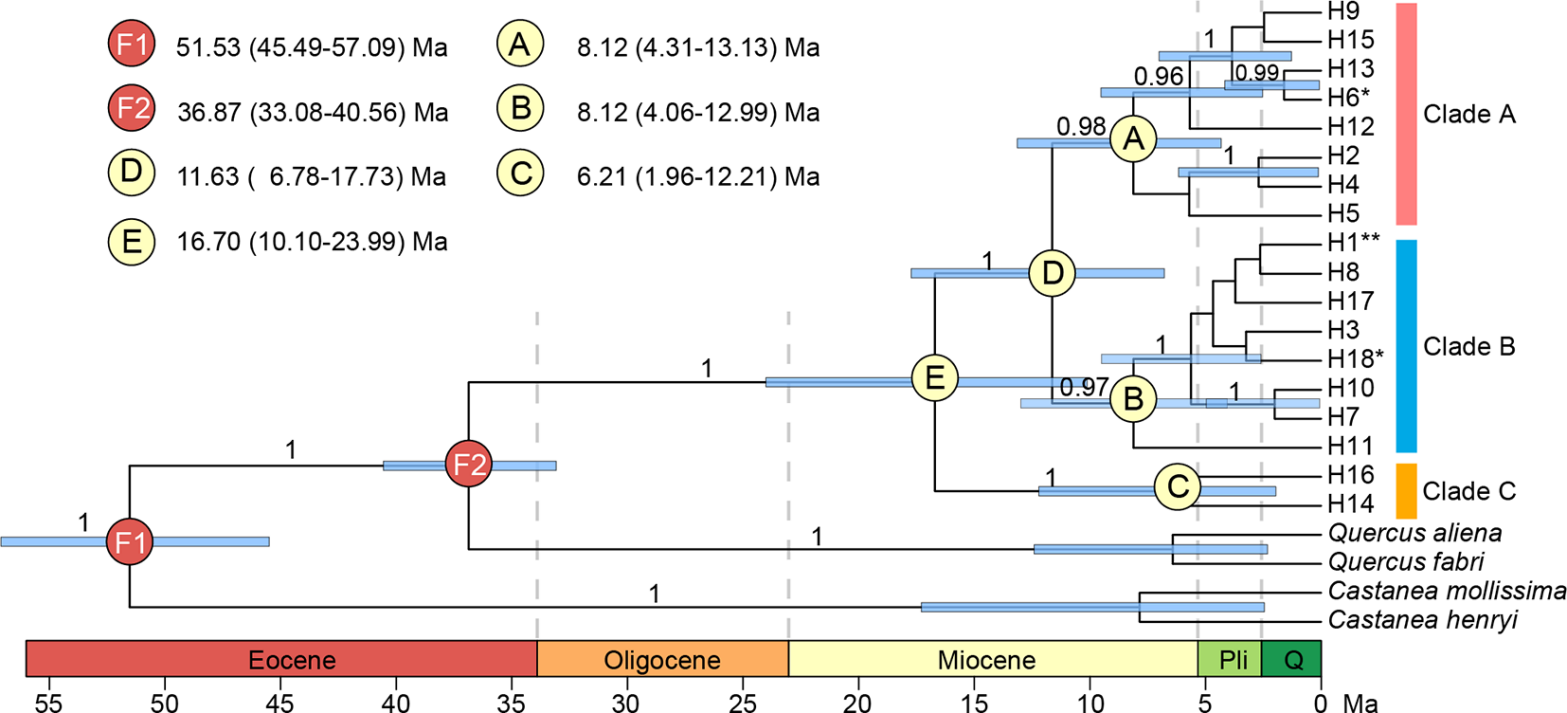
BEAST-derived chronograms for 18 chloroplast DNA haplotypes of *Quercus chenii*, with species from *Quercus* section *Quercus* and genus *Castanea* as outgroups. Blue bars indicate the 95% highest posterior density (HPD) credibility intervals for node ages (million years ago, Ma). Posterior probabilities (>0.9) are labeled above nodes. Geological time abbreviation: Pli, Pliocene; Q, Quaternary. **, shared by seven populations; *, shared by two populations.

Both median-joining network and phylogenetic analysis grouped all the 18 haplotypes into three clades (**Figures 6B** and **7**). Clade A included eight haplotypes that were geographically scattered in the Mufu-Jiuling-Lu mountain region, and the Jiuhua-Huangshan-Tianmu-Baiji-Huaiyu mountain region. Clade B was characterized by a notable star-like pattern, with ancestral haplotype H1 and seven derived haplotypes widely distributed in both northern and southern regions. Clade C contained only two haplotypes that were separated from clade B by five to seven mutations. Among those, H16 was only detected in population NJ, and H14 was confined to population ZN in the East Tianmu Mountains (**Figure 6B**). The time to the most recent common ancestor of all the haplotypes was dated to the early Miocene (16.70 Ma, 95% HPD: 10.10–23.99 Ma). Clade A and clade B were estimated to have diverged at the end of the middle Miocene (11.63 Ma, 95% HPD: 6.78–17.73 Ma). The crown ages of clade A, B, and C were about 8.12 Ma (95% HPD: 4.31–13.13 Ma), 8.12 Ma (95% HPD: 4.06–12.99 Ma), and 6.21 Ma (95% HPD: 1.96–12.21 Ma), respectively (**Figure 7**).

### Ecological Niche Modeling and Dispersal Corridors

**Figure 8:**
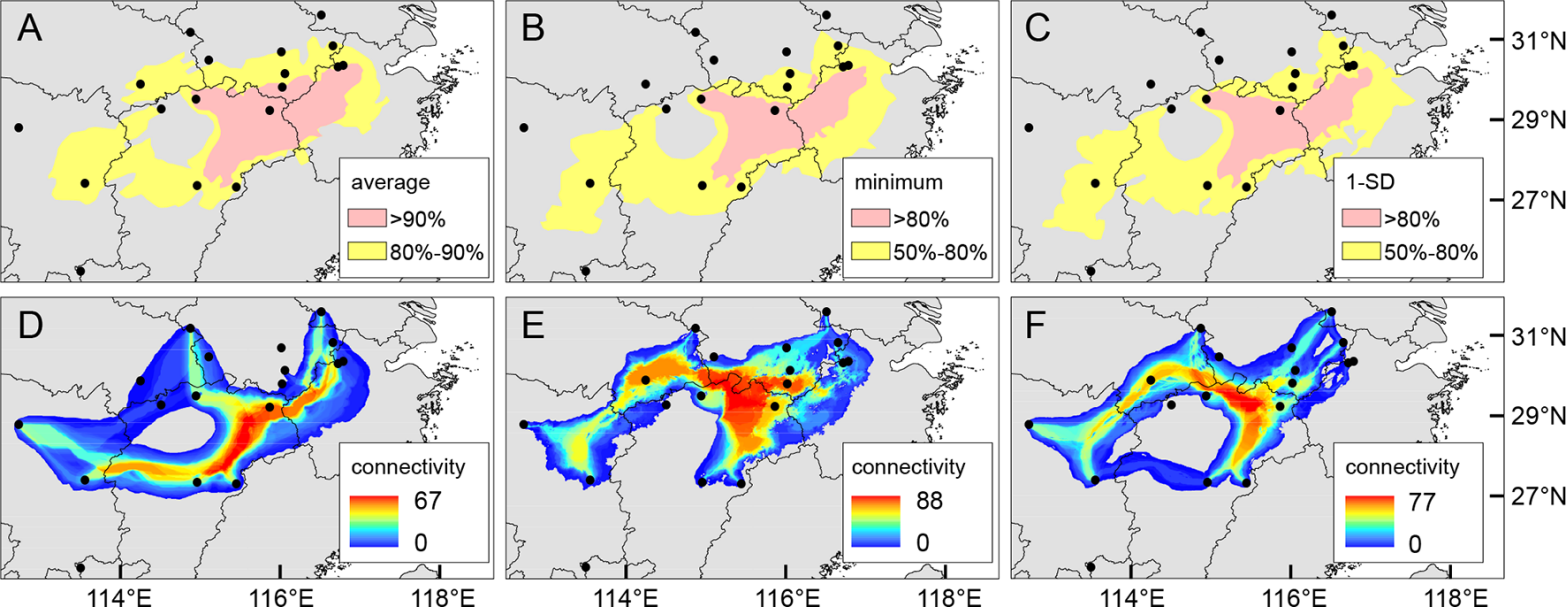
**(A–C)** Putative areas with moderate (yellow) or high (pink) ecological stability for *Quercus chenii* based on the average **(A)**, minimum **(B)**, and 1-SD **(C)** of occurrence probabilities across the Last Interglacial (LIG), the Last Glacial Maximum (LGM), the Mid Holocene, and the present estimated by MAXENT 3.4.1 (Phillips et al., 2018), using 80% and 90% (for average), and 50% and 80% (for minimum and 1-SD) of the maximum values as thresholds. **(D–F)** Potential dispersal corridors during the LGM **(D)**, the Mid Holocene **(E)**, and at the present **(F)** for *Q. chenii* estimated by SDMTOOLBOX (Brown, 2014). Black dots indicate the 18 sampling sites of this study.

The mean AUC value (± SD) on the test dataset was 0.921 ± 0.031, indicating a good fit of ENM to the observed occurrence data of *Q. chenii*. Historical distributions for the LIG, LGM, and Mid Holocene are shown in **Supplementary Figure S7**. An area with high ecological stability in the mountainous area of East China was identified by calculating the average, minimum, and 1-SD of occurrence probabilities across different periods (**Figures 8A–C**). A major north–south dispersal corridor along the plains and hills on the eastern side of the Poyang Lake Basin was detected for the LGM, the Mid Holocene, and the present. Two east–west dispersal corridors along the hills in the central Jiangxi Province and north of the Mufu Mountains were also identified for the LGM and the present, respectively (**Figures 8D–F**).

## Discussion

### Mountains Facilitated Population Differentiation Through Both Geographic Isolation and Environmental Isolation

Our current study of *Q. chenii* indicated that a higher level of genetic differentiation occurred among highland populations than among lowland populations (*P* < 0.001, **Table 2**). A more detailed analysis using BARRIER identified multiple genetic boundaries that were well associated with the scattered distribution of mountain ranges in East China (**Figure 1B**). Such findings pinpointed that mountains as landscape barriers may have driven the population differentiation through long-term geographic isolation. The occurrence of multiple geographic barriers was also supported by the distribution pattern of cpDNA haplotypes (**Figure 6**). Specifically, no shared haplotypes were detected between populations inside and outside the region surrounded by the Mufu Mountains, the Jiuling Mountains, and the Lu Mountains, and the region surrounded by the Jiuhua Mountains, the Huangshan Mountains, the Tianmu Mountains, the Baiji Mountains, and the Huaiyu Mountains. More strikingly, five of the seven haplotypes within these two mountainous regions were private to a single population. This pattern was more likely to result from long-term *in situ* diversification among isolated montane habitats. Considering the limited seed-mediated gene flow for oaks, differentiation may be enhanced by stochastic factors such as genetic drift (Zhang et al., 2013). Similar patterns with a geographic mosaic of cpDNA haplotypes have also been observed for several wind-pollinated tree species amid the complex topography in subtropical China, e.g., *Fagus lucida* (Zhang et al., 2013), *Fagus longipetiolata* (Zhang et al., 2013), and *Juglans cathayensis* (Bai et al., 2014), suggesting that the persistent landscape barriers have played an important role in shaping the pattern of long-term isolation for the extant plant species in this region.

In addition to geographic isolation, mountains may steepen climatic gradients, and further facilitate population differentiation through environmental isolation (Ohsawa and Ide, 2008; Sexton et al., 2016). For wind-pollinated trees like oaks, the pollen-mediated gene flow is not likely to be constrained by discontinuous habitats sharing similar environmental conditions (Wanderley et al., 2018), but can be disrupted by environmental dissimilarity (Sexton et al., 2014). Thus, a significant pattern of IBE would be expected for wind-pollinated plants, which has been observed for ash (Temunović et al., 2012), larch (Nishimura and Setoguchi, 2011), and also several oak species in East Asia (*Q. liaotungensis*, Yang et al., 2018) and North America (*Q. lobata*, Gugger et al., 2013; *Q. engelmannii*, *Q. berberidifolia*, *Q. cornelius-mulleri*, Riordan et al., 2016). Using redundancy analysis for nuclear microsatellite data, we revealed that both IBE and IBD explained similar amounts of constrained genetic variation across the species’ distribution, and elevation contributed the most to explaining the genetic variation constrained by RDA axis 1 (**Figure 5** and **Supplementary Table S7**), highlighting that environmental isolation associated with the complex topography in East China was also responsible for the local patterns of neutral microsatellite variation of *Q. chenii*.

The genetic differentiation along altitudinal gradients was also supported by Bayesian cluster analysis, which assigned a much lower proportion of genetic cluster III to the high elevation populations (**Figure 1A**). Multiple clues can be integrated to explain these findings. First, during the fieldwork, an elevation-related difference was observed regarding vegetation cover and topographic heterogeneity. Specifically, most individuals of lowland populations were found to be accompanied by short shrubs or sparse deciduous trees on plains and hills (**Figure 1C**), whereas most individuals of highland populations are scattered in thick forests, which are dominated by evergreen broad-leaved trees like *Castanopsis eyrei* and *Quercus glauca*, and have a continuous distribution across mountains and valleys (**Figure 1D**). Such a difference may have led to asymmetric pollen gene flow, and further reduced the genetic connectivity between highland and lowland populations (Gharehaghaji et al., 2017). Second, microclimate is also associated with elevation. Highland habitats are characterized by significantly lower temperature of spring, and a wetter environment surrounded by montane landscapes (Wang et al., 2010). These factors can influence the asynchronism of reproductive phenology, e.g., the timing of flowering and pollen release (Montoya-Pfeiffer et al., 2016), and then increase the effects of IBE through nonrandom mating due to climatic differences along altitudinal gradients. Finally, highland populations exhibited stronger differentiation and much lower within-population diversity than those in lowland areas (**Table 2** and**** **Figure 3**), suggesting that the species may have experienced genetic drift and long-term fragmentation among multiple island-like highland habitats, probably a consequence of historical colonization along altitudinal gradients and following ecological isolation (Qiu et al., 2009a; Wang et al., 2017).

Complementary to elevation, other environmental variables, such as temperature seasonality, annual mean temperature, and precipitation of warmest quarter, also contributed to the geographic patterns of climatically structured genetic variation significantly (**Supplementary Table S7**). Previous studies have shown that both temperature and precipitation factors can affect the timing of reproductive phenophases of plants, including oaks (Matthews and Mazer, 2016; Gerst et al., 2017). Thus, nonrandom mating due to asynchronism of reproductive phenology may be a critical driver that shapes the pattern of environmental isolation for *Q. chenii*. More investigations on gene movement, natural selection, and local adaption to different ecological processes are expected to provide a better understanding of the pattern of genetic variation for *Q. chenii*.

### Lowlands as Dispersal Corridors Increased the Genetic Connectivity Among Populations When the Species Experienced Range Expansions

Contrary to the role of mountains as geographic barriers, lowlands are more likely to be dispersal corridors for *Q. chenii*. Using least-cost path calculations, we confirmed that a major south–north dispersal corridor occurred along the plains and hills on the eastern side of the Poyang Lake Basin, and two east–west dispersal corridors occurred along the hills in the central Jiangxi Province and north of the Mufu Mountains (**Figures 8D–F**). Our investigations supported the conclusions of Fan et al. (2017), which showed that stronger population connectivity occurred in the eastern Poyang Lake Basin corridor for five plant species in subtropical China, and connections also occurred between the eastern and the western populations across the basin. However, the corridors detected in this study occupied a much lower elevation than those identified by Fan et al. (2017) (e.g., the Wuyi Mountains and the Yushan Mountains), implying that lowlands are important for the migration of plants that prefer a habitat at low elevation regions.

Furthermore, although the mismatch distribution analysis, Tajima’s *D* and Fu’s *F*_S_ tests did not show strong evidence of extensive demographic expansion, the star-like cpDNA phylogeny of clade B and the widespread distribution of the ancestral haplotype H1 suggested that lowland populations could have experienced local expansions during the interglacial periods. These results indicated that lowlands may have contributed to the post-glacial south-to-north range shifts of *Q. chenii*. The species may have had a southern refugium, probably at the locations of populations LC and LA that harbored the ancestral haplotype H1. During the interglacial periods, *Q. chenii* may have migrated northward to the region north of the Yangtze River along the dispersal corridors identified in this study.

The role of lowlands as dispersal corridors was also supported by the evidence inferred from nuclear microsatellite variation. The general linear model showed that elevation best explained the spatial pattern of genetic admixture across the species’ range (**Table 5**). Compared with highland populations, lowland populations exhibited a higher degree of genetic admixture, but a lower level of population differentiation (**Table 2**). These results suggested the landscape features in East China play different roles in shaping the patterns of gene flow within the species. Mountains as geographic barriers may have reduced gene exchanges among isolated habitats, and led to stronger patterns of both IBD and IBE. By contrast, lowlands were more likely to be dispersal corridors, which were critical for the post-glacial colonization, and contributed to the increased genetic admixture due to the arrival of immigrants originating from genetically differentiated populations (Ortego et al., 2015). To further support this hypothesis, we performed the ABC analysis to reconstruct the past demographic history of *Q. chenii*. The results clearly favoured the scenario that the lowland populations of *Q. chenii* may have experienced a recent expansion during the LIG, which is in agreement with the much larger potential distribution predicted by ENMs for this period (**Supplementary Figure S7A**). Overall, both cpDNA and nSSR markers showed evidence that lowlands were more likely to be dispersal corridors for *Q. chenii*, especially during the periods of range expansions. However, due to different effective population sizes and dispersal capabilities for nuclear and chloroplast genomes, they responded to topographic and climatic factors in different ways. Seed-mediated gene flow of oaks is more likely to be restricted by habitat fragmentation, and cannot counteract the effects of genetic drift (Petit et al., 1997). Thus, we detected shared haplotypes among lowland populations, but without much variation within populations. By contrast, pollen gene flow is much stronger than genetic drift, together with the higher migration rate, it would increase the genetic connectivity among regions, and also result in a higher level of both admixture and within-population diversity for lowland populations.

### Lineage Divergence During the Neogene and Quaternary Refugia in a Mountainous Area of East China

Accumulating evidence has shown that the tectonic–climatic interactions since the Miocene have significantly influenced the evolutionary dynamics of plant species in East Asia (e.g., Kou et al., 2015; Yu et al., 2017; Deng et al., 2018). Similar to prior studies, we conclude that the lineage divergence of *Q. chenii* has been shaped by the palaeoclimatic changes of the Neogene. The dating analyses of major cpDNA clades suggested that *Q. chenii* may have experienced the earliest divergence during the early Miocene (node E, 16.70 Ma, 95% HPD: 10.10–23.99 Ma, **Figure 7**). This age is more recent than the earliest unambiguous fossil records of *Quercus* section *Cerris* from the early Oligocene of Russia Far East (Denk et al., 2017), but close to the first occurrence of the reliable fruit fossils that were morphologically closely related to extant oak species from the section (i.e., *Q. acutissima* and *Q. variabilis*) in China (Song et al., 2000), implying a probable early Miocene origin of *Q. chenii* and its siblings. The estimated time here is also comparable with the crown ages of other tree species endemic to subtropical China, such as *Cyclocarya paliurus* (16.69 Ma, 95% HPD: 8.42–27.86 Ma, Kou et al., 2015), and dominant woody genera of subtropical evergreen broadleaved forests in East Asia, such as *Machilus* (15.8 Ma, 95% HPD: 9.9–21.6 Ma, Li et al., 2011), *Castanopsis* (17.7 Ma, 95% HPD: 11.5–24.7 Ma, Xing et al., 2014), and *Michelia* (19.2 Ma, 95% HPD: 13.2–25.9 Ma, Nie et al., 2008), suggesting that the initiation, or more likely, the intensification of the East Asian monsoon system (Sun and Wang, 2005; Licht et al., 2014), and subsequent increased humidification in the southern China (Guo et al., 2008) around the Oligocene–Miocene boundary may have triggered the lineage divergence of *Q. chenii* and other plant species of subtropical evergreen broadleaved forests in East Asia (Yu et al., 2017; Deng et al., 2018).

The time-calibrated phylogeny indicated that the divergence between clade C and D was likely to have occurred at the boundary of the middle and the late Miocene (11.63 Ma, **Figure 7**). Three major subclades A–C were estimated to have diverged during the late Miocene (8.12–6.21 Ma, **Figure 7**). Moreover, most haplotypes were found to have diverged during the late Pliocene to the early Pleistocene (3.86–1.60 Ma, **Figure 7**). Such findings were largely in agreement with the global climatic cooling after the Middle Miocene Climatic Optimum (17–15 Ma; Sun and Zhang, 2008), and the enhancement of East Asian monsoon intensity around 8–7 Ma and 3.5–1.6 Ma (An et al., 2001; Sun and Wang, 2005). Similar divergence history has also been reported for several woody taxa in East Asia, such as *Cyclocarya paliurus* (Kou et al., 2015), *Quercus* section *Cyclobalanopsis* (Deng et al., 2018), and Theaceae (Yu et al., 2017). More interestingly, a recent research also provided quantitative evidence to support the positive association of diversification rates with both global cooling and East Asian monsoon activity for *Primulina*, one of the most diverse herbaceous genera amid the complex karst landscapes in southern China (Kong et al., 2017). The combination of these findings pinpoints that the Neogene climate dynamics, especially the phased intensification of East Asian monsoons and global cooling of the Miocene, have stimulated the lineage divergence of plants in this region no matter whether they are herbs or long-lived trees.

Our analyses suggested that an area surrounded by multiple geographic barriers, i.e., the Tianmu Mountains, the Huangshan Mountains, the Baiji Mountains, and the Huaiyu Mountains, may have acted as a refugium for *Q. chenii*. This hypothesis was supported by three lines of evidence. First, ENM indicated that this area was characterized by moderate to high ecological stability throughout the LIG to the present (**Figures 8A–C**), implying that *Q. chenii* probably have survived *in situ* during the glacial periods of the Pleistocene. Second, a higher level of phylogenetic diversity was detected here. Although most populations were fixed for a single haplotype, all the three cpDNA haplotype clades, including several endemic haplotypes were concentrated, and populations dominated by the three genetic clusters identified by Bayesian cluster analysis were also confined to this area. Finally, macrofossils suggested that *Q. chenii* went extinct in central Japan along with the onset of glaciation in the early Pleistocene (Momohara, 2016), and probably retreated southward to the refugia on the East China Sea shelf (Li et al., 2016; **Supplementary Figure S7B**), and then to the refugia in Mainland China. Palaeovegetation reconstruction of the LGM also supported that temperate deciduous trees may have confined to the low elevation regions of East China and extended to the offshore refugia on the continental shelf (Harrison et al., 2001). Thus, the combined evidence of long-term climatic stability, higher phylogenetic diversity, and a location southwest of Japan and close to East China sea land bridge suggested that this region may have served as a mainland refugium for *Q. chenii*. Recent studies also revealed that plants with a larger distribution may have survived in a refugium located at eastern subtropical China (e.g., Wang et al., 2017; Hohmann et al., 2018). Our inference strengthened this belief and confirmed that the tree species mainly distributed in East China may have persisted *in situ*, and more strikingly, have been isolated for a long term among multiple habitats within the mountainous area. However, caution is still needed, given that several haplotypes detected in this study were shared with a closely related species, *Q. acutissima* (Li et al., in press), although the same region may also have been a refugium for the latter, implying a probable scenario of interspecific introgression due to secondary contact or long-term co-occurrence in a shared refugium.

## Conclusions

Our study illustrates that the pattern of genetic variation of *Q. chenii* was strongly influenced by both topography and climate. Palaeoclimatic changes of the Miocene may have driven the lineage divergence of chloroplast haplotypes. Persistent landscape barriers in East China may have facilitated population differentiation through both long-term geographic isolation and environmental isolation along altitudinal and other climatic gradients. By contrast, post-glacial range shifts along plains and basins may have increased the genetic connectivity among lowland populations *via* admixture.

## Author Contributions

YL and YF conceived and designed this research. YL and XZ collected samples and performed experiments. YL analyzed the data. YL led writing with substantial contributions from YF. All authors read and approved the final manuscript.

## Funding

This research was financially supported by the National Natural Science Foundation of China (31770699, 31370666), the Priority Academic Program Development of Jiangsu Higher Education Institutions (PAPD), and the Postgraduate Research and Practice Innovation Program of Jiangsu Province (KYLX15_0922).

## Conflict of Interest Statement

The authors declare that the research was conducted in the absence of any commercial or financial relationships that could be construed as a potential conflict of interest.
