# Supplementary material for: Landscape Features and Climatic Forces Shape the Genetic Structure and Evolutionary History of an Oak Species (Quercus chenii) in East China
Source: Front Plant Sci. 2019 Sep 3;10:1060. doi: 10.3389/fpls.2019.01060 (PMC6734190; doi:10.3389/fpls.2019.01060)

**Supplementary Figure S2** Linear regression between elevation and the probability of membership (*Q*) to genetic clusters I (*Q*_I_), II (*Q*_II_), and III (*Q*_III_) in each population of *Quercus chenii* when *K* = 3.


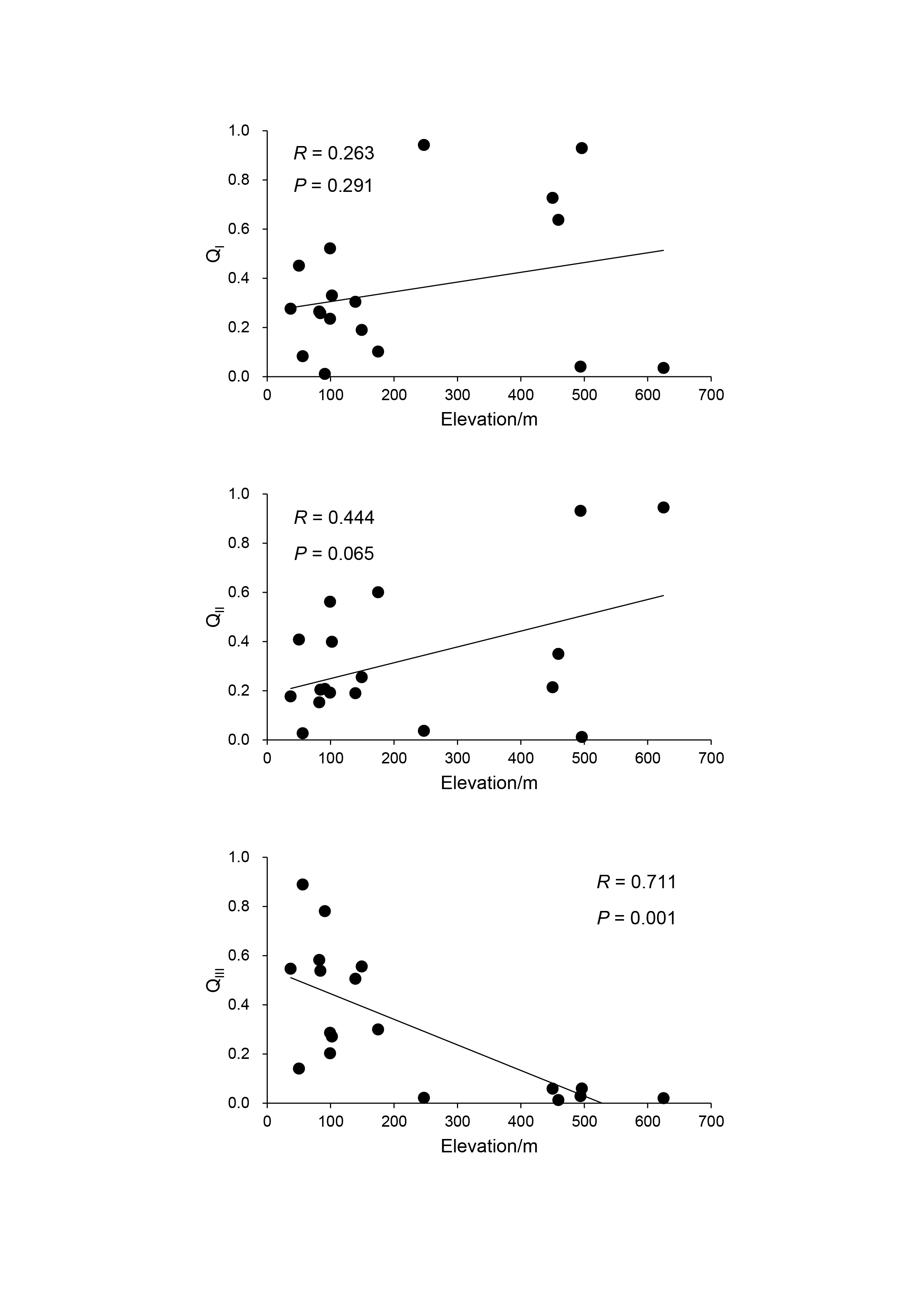

Supplement: Supplementary file 1 [file DataSheet_1.zip › Figure_S2.docx]
