# Supplementary material for: Landscape Features and Climatic Forces Shape the Genetic Structure and Evolutionary History of an Oak Species (Quercus chenii) in East China
Source: Front Plant Sci. 2019 Sep 3;10:1060. doi: 10.3389/fpls.2019.01060 (PMC6734190; doi:10.3389/fpls.2019.01060)
Supplement: Supplementary file 1 [file DataSheet_1.zip › Figure_S3.pdf]

Supplementary Figure S3 Bar plots of individual membership coefficients for each value of *K*, and each mode within 20 total runs of a given *K*. Each vertical bar represents one individual.

Major modes for the uploaded data:

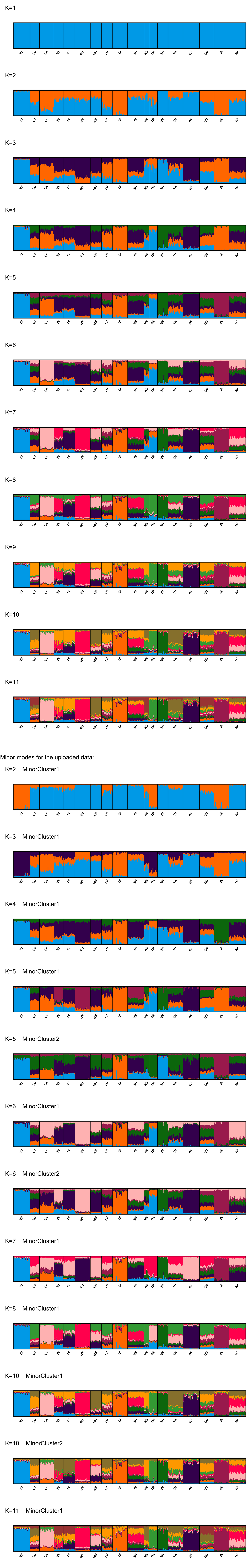

Division of runs by mode:

K=1 20/20
K=2 17/20, 2/20
K=3 13/20, 7/20
K=4 10/20, 10/20
K=5 15/20, 2/20, 2/20
K=6 13/20, 3/20, 3/20
K=7 15/20, 4/20
K=8 18/20, 2/20
K=9 20/20
K=10 12/20, 4/20, 4/20
K=11 17/20, 2/20
