# Supplementary material for: Landscape Features and Climatic Forces Shape the Genetic Structure and Evolutionary History of an Oak Species (Quercus chenii) in East China
Source: Front Plant Sci. 2019 Sep 3;10:1060. doi: 10.3389/fpls.2019.01060 (PMC6734190; doi:10.3389/fpls.2019.01060)

**Supplementary Figure S4** Principal component analysis (PCA) based on four summary statistics to check the goodness of fit of scenario 4 for both highland (A) and lowland (B) populations of *Quercus chenii*.


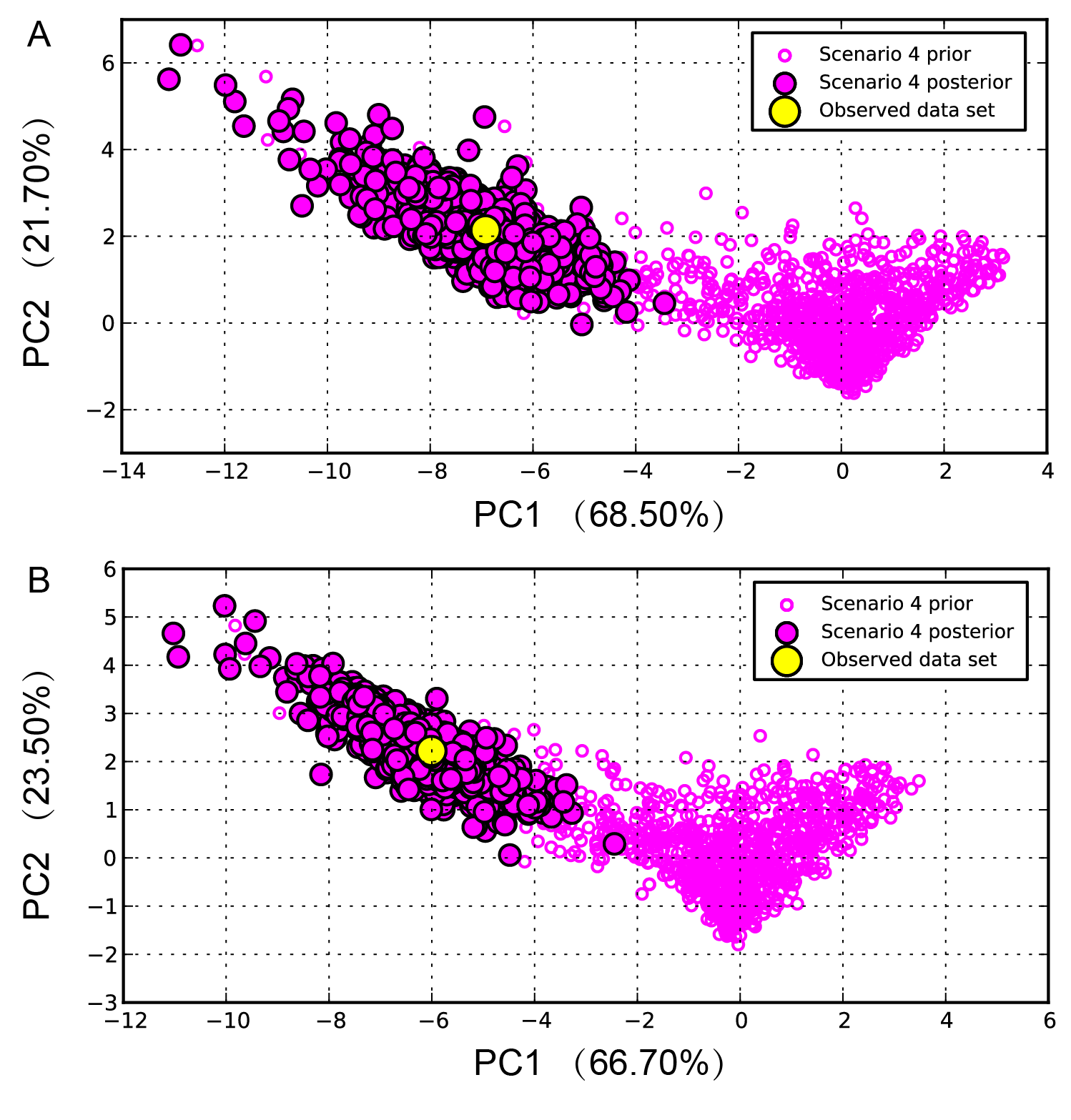

Supplement: Supplementary file 1 [file DataSheet_1.zip › Figure_S4.docx]
