# Supplementary material for: Landscape Features and Climatic Forces Shape the Genetic Structure and Evolutionary History of an Oak Species (Quercus chenii) in East China
Source: Front Plant Sci. 2019 Sep 3;10:1060. doi: 10.3389/fpls.2019.01060 (PMC6734190; doi:10.3389/fpls.2019.01060)

**Supplementary Figure S5** Prior (red line) and posterior (green line) distributions of current effective population size (*N*_1_), effective population size before the expansion event (*N*_3_), and time of the expansion event in generations (*t*_1_) for both highland and lowland populations of *Quercus chenii* in scenario 4.


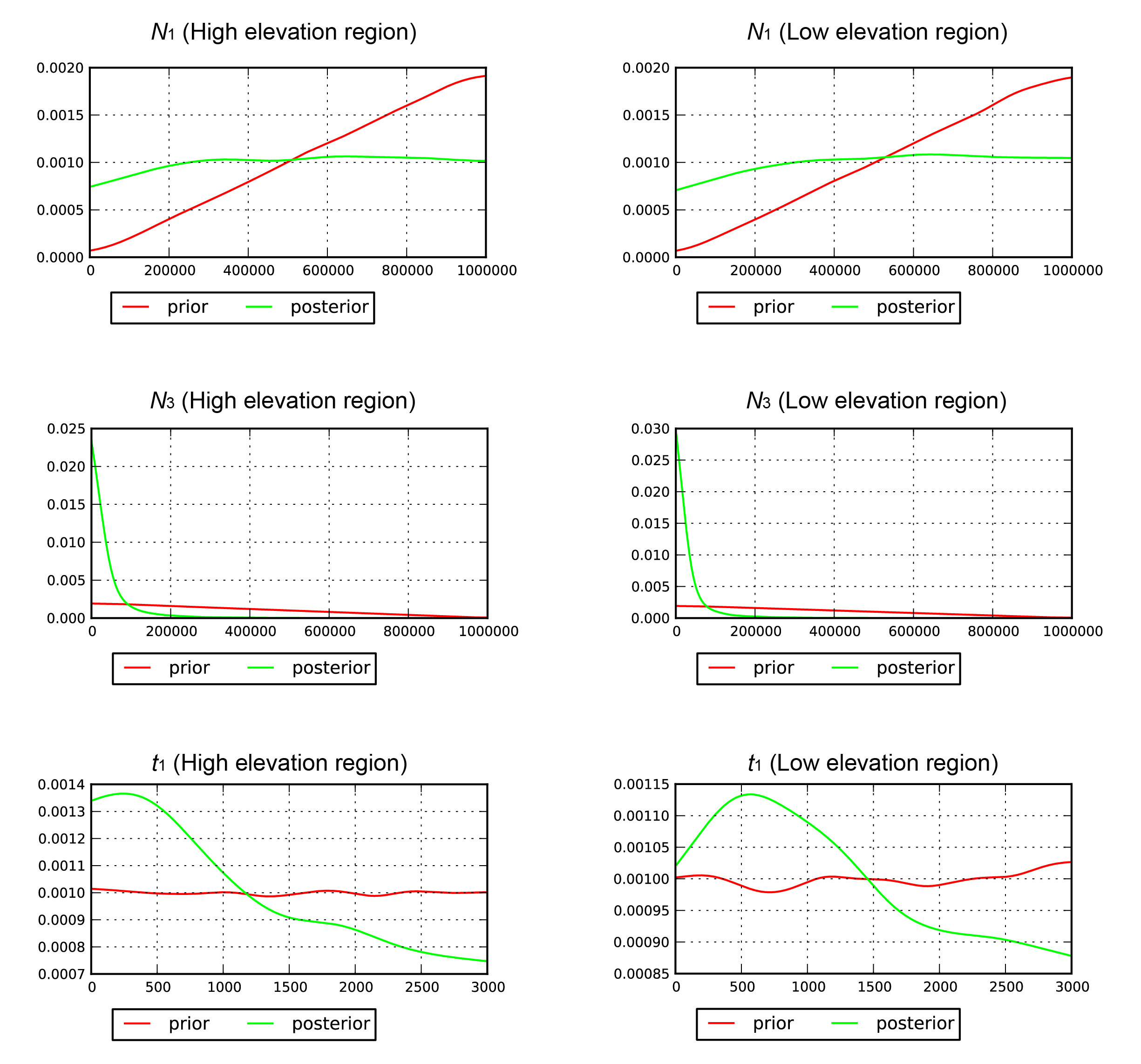

Supplement: Supplementary file 1 [file DataSheet_1.zip › Figure_S5.docx]
