# Supplementary material for: Landscape Features and Climatic Forces Shape the Genetic Structure and Evolutionary History of an Oak Species (Quercus chenii) in East China
Source: Front Plant Sci. 2019 Sep 3;10:1060. doi: 10.3389/fpls.2019.01060 (PMC6734190; doi:10.3389/fpls.2019.01060)

**Supplementary Figure S7** Potential distributions of the Last interglacial (A), the Last Glacial Maximum (B), the Mid Holocene (C), and present (D) for *Quercus chenii* estimated by MAXENT. Black and green dots indicate sampling sites and occurrence records used in this study.


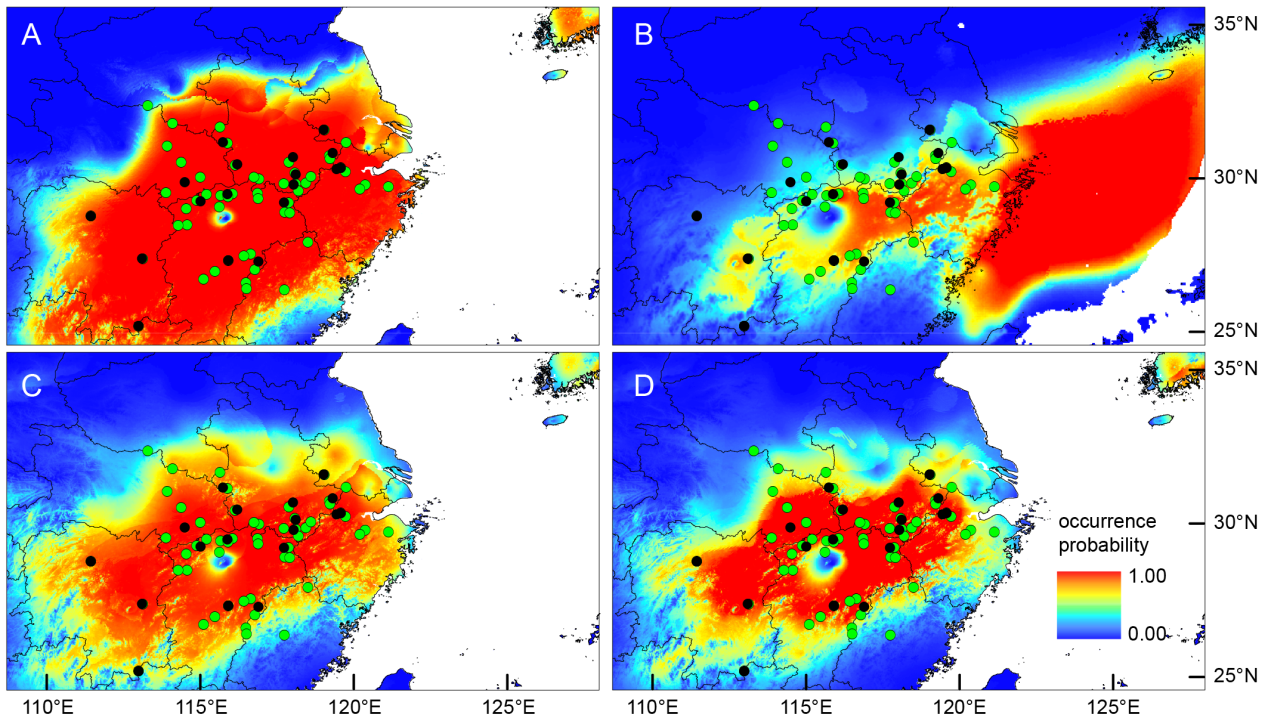

Supplement: Supplementary file 1 [file DataSheet_1.zip › Figure_S7.docx]
