# Supplementary material for: Landscape Features and Climatic Forces Shape the Genetic Structure and Evolutionary History of an Oak Species (Quercus chenii) in East China
Source: Front Plant Sci. 2019 Sep 3;10:1060. doi: 10.3389/fpls.2019.01060 (PMC6734190; doi:10.3389/fpls.2019.01060)
Supplement: Supplementary file 1 [file DataSheet_1.zip › Table_S1.docx]

**Supplementary Table S1** Locations, collection numbers and collectors for samples of 18 populations of *Quercus chenii* in East China.

| Population code | Location | Longitude (°E) | Latitude (°N) | Collection numbers | Collectors |
| --- | --- | --- | --- | --- | --- |
| **Highland populations** | | | |  |  |
| YZ | Liyuan Town, Yizhang, Hunan, China | 112.99 | 25.21 | YL_YZ_01-YL_YZ_30 | Yao Li, Qingliang Liu |
| HS | Huangshan Mountain, Huangshan, Anhui, China | 118.11 | 30.14 | YL_HS_01-YL_HS_09 | Yao Li, Baokun Xu, Lu Wang |
| TM | West Tianmu Mountain, Lin'an, Zhejiang, China | 119.44 | 30.32 | YL_TM_01-YL_TM_15 | Yao Li, Baokun Xu, Xuan Li |
| QI | Qiyun Mountain, Xiuning, Anhui, China | 118.04 | 29.81 | YL_QI_01-YL_QI_27 | Yao Li, Jianping Deng |
| ZN | Zhinan Village, Lin'an, Zhejiang, China | 119.57 | 30.36 | YL_ZN_01-YL_ZN_19 | Yao Li, Kaiwen Zhang |
| JZ | Tiantangzhai Town, Jinzhai, Anhui, China | 115.74 | 31.19 | YL_JZ_01-YL_JZ_27 | Yao Li, Zenglin Cheng |
| **Lowland populations** | | | |  |  |
| XN | Gaoqiao Town, Xianning, Hubei, China | 114.49 | 29.89 | YL_XN_01-YL_XN_30 | Yao Li, Linhong Hu |
| GD | Baidian Town, Guangde, Anhui, China | 119.31 | 30.84 | YL_GD_01-YL_GD_26 | Yao Li |
| QY | Zhaohua Village, Qingyang, Anhui, China | 118.02 | 30.70 | YL_QY_01-YL_QY_30 | Yao Li, Xingwang Zhang |
| WN | Futian Township, Wuning, Jiangxi, China | 115.01 | 29.27 | YL_WN_01-YL_WN_20 | Yao Li |
| ZZ | Zhuanqiao Town, Zhuzhou, Hunan, China | 113.10 | 27.40 | YL_ZZ_01-YL_ZZ_17 | Yao Li, Qingliang Liu |
| WY | Ziyang Town, Wuyuan, Jiangxi, China | 117.73 | 29.23 | YL_WY_01-YL_WY_28 | Yao Li, Baoyuan Zha |
| LU | Zhangjia Village, Lushan, Jiangxi, China | 115.88 | 29.50 | YL_LU_01-YL_LU_20 | Yao Li, Xingwang Zhang |
| TH | Tianhua Town, Taihu, Anhui, China | 116.20 | 30.48 | YL_TH_01-YL_TH_27 | Yao Li, Longjia Wang |
| LC | Rifeng Town, Lichuan, Jiangxi, China | 116.89 | 27.31 | YL_LC_01-YL_LC_17 | Yao Li, Yong Zhou |
| TY | Taohuayuan Town, Taoyuan, Hunan, China | 111.44 | 28.79 | YL_TY_01-YL_TY_21 | Yao Li, Qingliang Liu |
| NJ | Wuxiang Mountain, Lishui, Jiangsu, China | 119.02 | 31.61 | YL_NJ_01-YL_NJ_30 | Yao Li |
| LA | Nancun Township, Le'an, Jiangxi, China | 115.91 | 27.34 | YL_LA_01-YL_LA_26 | Yao Li, Guokang Peng |
