# Supplementary material for: Landscape Features and Climatic Forces Shape the Genetic Structure and Evolutionary History of an Oak Species (Quercus chenii) in East China
Source: Front Plant Sci. 2019 Sep 3;10:1060. doi: 10.3389/fpls.2019.01060 (PMC6734190; doi:10.3389/fpls.2019.01060)
Supplement: Supplementary file 1 [file DataSheet_1.zip › Table_S10.docx]

**Supplementary Table S10** Partitioning of the genetic variation of *Quercus chenii* purely associated with climate and geography using redundancy analyses (RDA) and partial RDA. The proportion of genetic variation that could not be partitioned due to the collinearity between geographic and climatic variables (climate + geography) is also reported.

| Genetic variation | Partitioned variance | Proportion constrained (%) | *P*-value |
| --- | --- | --- | --- |
| Total variance | 0.56644 |  |  |
| Full model | 0.07314 | 12.91 | 0.001 |
| Pure climate | 0.03454 | 6.10 | 0.001 |
| Pure geography | 0.03525 | 6.22 | 0.001 |
| Climate + geography | 0.00335 | 0.59 |  |
