# Supplementary material for: Landscape Features and Climatic Forces Shape the Genetic Structure and Evolutionary History of an Oak Species (Quercus chenii) in East China
Source: Front Plant Sci. 2019 Sep 3;10:1060. doi: 10.3389/fpls.2019.01060 (PMC6734190; doi:10.3389/fpls.2019.01060)
Supplement: Supplementary file 1 [file DataSheet_1.zip › Table_S11.docx]

**Supplementary Table S11** Eighteen haplotypes identified in this study based on 16 nucleotide substitutions and six indels.

|  | *atp*B-*rbc*L | | |  | *trn*H-*psb*A | | | | | | | |  | *trn*S-*trn*G | | | | | | | |  | *trn*S-*trn*T | | |
| --- | --- | --- | --- | --- | --- | --- | --- | --- | --- | --- | --- | --- | --- | --- | --- | --- | --- | --- | --- | --- | --- | --- | --- | --- | --- |
|  | 2  7  7 | 3  5  3 | 4  6  5 |  | 8  2  8 | 8  3  2 | 880  -887 | 914  -955 | 1  1  0  7 | 1133  -1150 | 1194 -1214 | 1  2  7  9 |  | 1  4  6  6 | 1  4  9  2 | 1  5  9  8 | 1632  -1638 | 1  6  4  6 | 1  8  3  2 | 1  8  9  4 | 1959  -1963 |  | 2  0  8  3 | 2  4  2  3 | 2  8  2  6 |
| H1 | T | G | A |  | T | C | I1 | — | C | — | — | A |  | C | T | C | I8 | T | G | A | I9 |  | T | C | C |
| H2 | T | A | A |  | T | C | I1 | — | C | — | I7 | A |  | C | G | C | — | T | G | A | I9 |  | T | C | A |
| H3 | T | G | A |  | T | C | I1 | — | C | — | — | A |  | C | T | C | I8 | T | G | A | I9 |  | G | C | C |
| H4 | T | A | A |  | T | C | I1 | — | C | — | I7 | A |  | C | G | C | — | T | G | A | I9 |  | T | C | C |
| H5 | T | A | A |  | T | C | I1 | — | C | — | — | A |  | C | G | C | — | T | A | A | I9 |  | T | C | C |
| H6 | T | A | A |  | T | C | I1 | I3 | C | I6 | — | A |  | T | G | C | I8 | T | G | A | I9 |  | T | C | C |
| H7 | T | G | A |  | T | C | I2 | — | C | — | — | A |  | C | T | C | I8 | T | G | A | I9 |  | T | C | C |
| H8 | T | G | A |  | G | C | I1 | — | C | — | — | A |  | C | T | C | I8 | T | G | A | I9 |  | T | C | C |
| H9 | T | A | A |  | T | C | I1 | I4 | C | — | — | A |  | C | G | C | I8 | T | G | A | I9 |  | T | C | C |
| H10 | T | G | A |  | T | C | I2 | I5 | C | — | — | A |  | C | T | C | I8 | T | G | A | I9 |  | T | C | C |
| H11 | T | G | A |  | T | C | I1 | — | C | — | — | C |  | C | T | C | — | T | G | G | I9 |  | T | C | C |
| H12 | T | A | G |  | T | C | I1 | — | C | — | — | A |  | C | G | C | I8 | T | G | A | — |  | T | C | C |
| H13 | T | A | A |  | T | C | I1 | I3 | C | — | — | A |  | T | G | C | I8 | T | G | A | I9 |  | T | C | C |
| H14 | T | G | A |  | T | T | — | — | C | — | — | A |  | C | G | T | I8 | G | G | A | I9 |  | T | G | C |
| H15 | T | A | A |  | T | C | I1 | I3 | C | — | — | A |  | C | G | C | I8 | T | G | A | I9 |  | T | C | C |
| H16 | T | G | A |  | T | T | — | — | T | — | — | A |  | C | T | C | I8 | G | G | A | I9 |  | T | C | C |
| H17 | T | G | A |  | T | C | I1 | — | C | — | — | A |  | C | T | C | I8 | T | G | A | I9 |  | T | C | A |
| H18 | C | G | A |  | T | C | I1 | — | C | — | — | A |  | C | T | C | I8 | T | G | A | I9 |  | T | C | C |

I1, ATTTATTA; I2, ATTTTTTA; I3, TTAATTATTAAATTATTATAT; I4, TTAATTATTAAATTATTATATTTAATTATTAAATTATTATAT; I5, TATTAAATTATTATAT; I6, TTTTTATTTATTTATTTA; I7, ATTTATTTTCTGCCTCTTGTC; I8, CTATAAA; I9, AGAAT.
