# Supplementary material for: Landscape Features and Climatic Forces Shape the Genetic Structure and Evolutionary History of an Oak Species (Quercus chenii) in East China
Source: Front Plant Sci. 2019 Sep 3;10:1060. doi: 10.3389/fpls.2019.01060 (PMC6734190; doi:10.3389/fpls.2019.01060)
Supplement: Supplementary file 1 [file DataSheet_1.zip › Table_S2.docx]

**Supplementary Table S2** Primer sequences, annealing temperatures (*T*_A_), and lengths of consensus sequences for the four chloroplast intergenic spacers used in this study.

| Primer | Primer sequence (5'-3') | *T*_A_ (°C) | Length (bp) | Reference |
| --- | --- | --- | --- | --- |
| *atp*B-*rbc*L | F:TAGTTTCTGTTTGTGGTGACAT R:AAGTAGTAGGATTGGTTCTCAT | 55 | 718 | Okaura et al., 2007 |
| *psb*A-*trn*H | F:ACGGGAATTGAACCCGCGCA R:TATTATTAACCGTGCTAACC | 57 | 639 | Okaura et al., 2007 |
| *trn*S(GCU)-  *trn*G(UCC) | F:GCCGCTTTAGTCCACTCAGC R:GAACGAATCACACTTTTACCAC | 60 | 607 | Hamilton, 1999 |
| *trn*S(GCU)-  *trn*T(GGU) | F:GAGATGGCCGAGTGGTTGAA R:CCCGCTTAGCTCAGAGGTTAGAG | 60 | 902 | Kanno et al., 2004 |

Hamilton, M. B. (1999). Four primer pairs for the amplification of chloroplast intergenic regions with intraspecific variation. *Mol. Ecol* 8, 521–523.

Kanno, M., Yokoyama, J., Suyama, Y., Ohyama, M., Itoh, T., and Suzuki, M. (2004). Geographical distribution of two haplotypes of chloroplast DNA in four oak species (*Quercus*) in Japan. *J. Plant Res.* 117, 311–317. doi: 10.1007/s10265-004-0160-8

Okaura, T., Quang, N. D., Ubukata, M., and Harada, K. (2007). Phylogeographic structure and late Quaternary population history of the Japanese oak *Quercus mongolica* var. *crispula* and related species revealed by chloroplast DNA variation. *Genes Genet. Syst.* 82, 465–477. doi: 10.1266/ggs.82.465
