# Supplementary material for: Landscape Features and Climatic Forces Shape the Genetic Structure and Evolutionary History of an Oak Species (Quercus chenii) in East China
Source: Front Plant Sci. 2019 Sep 3;10:1060. doi: 10.3389/fpls.2019.01060 (PMC6734190; doi:10.3389/fpls.2019.01060)
Supplement: Supplementary file 1 [file DataSheet_1.zip › Table_S3.docx]

**Supplementary Table S3** Primer sequences, annealing temperatures (*T*_A_), and genetic statistics for the 14 nuclear microsatellite (nSSR) loci used in this study. ** *P* < 0.001. Statistical significance is based on 10,000 permutations as implemented in MSA.

| Locus | Primer sequence  (5'-3') | *T*_A_ (°C) | Null | *F*_ST_ | *G'*_ST_ |
| --- | --- | --- | --- | --- | --- |
| quru-GA-0M07 | F: TTTAGCATCACATTTCCGTT R: TTTTGTGTCATCCGGTATTA | 52 | 0.01 | 0.040** | 0.082 |
| quru-GA-1H14 | F: GCTTGGGCTTGTTCCTACT R: CAACACTTCTCATGGATTAGAGA | 58 | 0.03 | 0.041** | 0.332** |
| quru-GA-1M17 | F: GTTTGTGCTTGCTGGGAGG R: TTCTTCTTAGCTTCCCAACTGAA | 58 | 0.01 | 0.055** | 0.321** |
| quru-GA-2G07 | F: GCCAACAAATTTAACTATCCAT R: TAACTGGGCTAGATAATCAG | 52 | 0.01 | 0.065** | 0.347** |
| ssrQrZAG4 | F: CGTCTATAAGTTCTTGGGTGA R: GTAACTATGATGTGATTCTTACTTCA | 54 | 0.02 | 0.060** | 0.357** |
| ssrQrZAG7 | F: CAACTTGGTGTTCGGATCAA R: GTGCATTTCTTTTATAGCATTCAC | 56 | 0.00 | 0.056** | 0.289** |
| ssrQrZAG11 | F: CCTTGAACTCGAAGGTGTCCTT R: GTAGGTCAAAACCATTGGTTGACT | 56 | 0.01 | 0.040** | 0.061 |
| ssrQrZAG59 | F: AACAATGAACTCATCAATCCAACA R: GCCATTGAAACTCTCGACCTCTT | 54 | 0.01 | 0.060** | 0.477** |
| ssrQrZAG96 | F: CCCAGTCACATCCACTACTGTCC R: GGTTGGGAAAAGGAGATCAGA | 58 | 0.01 | 0.042** | 0.186** |
| ssrQrZAG112 | F: TTCTTGCTTTGGTGCGCG R: GTGGTCAGAGACTCGGTAAGTATTC | 50 | 0.01 | 0.061** | 0.239** |
| ssrQpZAG15 | F: CGATTTGATAATGACACTATGG R: CATCGACTCATTGTTAAGCAC | 54 | 0.01 | 0.054** | 0.441** |
| ssrQpZAG110 | F: GGAGGCTTCCTTCAACCTACT R: GATCTCTTGTGTGCTGTATTT | 56 | 0.01 | 0.069** | 0.620** |
| QM57-3M | F: TGAGGAGGTTGGTGGAGAA R: ATGTGGAGGGCTAAGATTT | 52 | 0.01 | 0.036** | 0.069 |
| QM67-3M1 | F: TGGCTTATCCAATGTTTGTGATT R: GCGTCGGTGGCGGCTTAGAGATT | 54 | 0.04 | 0.055** | 0.195** |
| overall |  |  | 0.01 | 0.054** | 0.228** |

Null, mean null allele frequency; *F*_ST_, genetic differentiation index; *G'*_ST_, standardized genetic differentiation index
