# Supplementary material for: Landscape Features and Climatic Forces Shape the Genetic Structure and Evolutionary History of an Oak Species (Quercus chenii) in East China
Source: Front Plant Sci. 2019 Sep 3;10:1060. doi: 10.3389/fpls.2019.01060 (PMC6734190; doi:10.3389/fpls.2019.01060)
Supplement: Supplementary file 1 [file DataSheet_1.zip › Table_S4.docx]

**Supplementary Table S4** Four summary statistics used in DIYABC for checking the goodness of fit for scenario 4.

| Summary  statistics | Highland populations | |  | Lowland populations | |
| --- | --- | --- | --- | --- | --- |
|  | Observed value | Proportion  (simulated<  observed) |  | Observed value | Proportion  (simulated<  observed) |
| Mean number of alleles | 10.14 | 0.52 |  | 13.21 | 0.52 |
| Mean genic diversity | 0.67 | 0.51 |  | 0.71 | 0.53 |
| Mean size variance | 6.61 | 0.32 |  | 7.54 | 0.34 |
| Mean Garza-Williamson's M | 0.79 | 0.48 |  | 0.89 | 0.44 |
