# Supplementary material for: Landscape Features and Climatic Forces Shape the Genetic Structure and Evolutionary History of an Oak Species (Quercus chenii) in East China
Source: Front Plant Sci. 2019 Sep 3;10:1060. doi: 10.3389/fpls.2019.01060 (PMC6734190; doi:10.3389/fpls.2019.01060)
Supplement: Supplementary file 1 [file DataSheet_1.zip › Table_S5.docx]

**Supplementary Table S5** Prior distributions of the 11 parameters used in DIYABC.

| Parameter | Prior distribution | Minimum | Maximum |
| --- | --- | --- | --- |
| ***Effective population size*** |  |  |  |
| *N*_1_ | Uniform | 10 | 1 × 10^6^ |
| *N*_2_ (*N*_1_ < *N*_2_) | Uniform | 10 | 1 × 10^6^ |
| *N*_3_ (*N*_3_ < *N*_1_) | Uniform | 10 | 1 × 10^6^ |
| *N*_4_ (*N*_4_ < *N*_2_) | Uniform | 10 | 1 × 10^6^ |
| *N*_5_ (*N*_3_ < *N*_5_) | Uniform | 10 | 1 × 10^6^ |
| ***Time scale in generations*** |  |  |  |
| *t*_1_ | Uniform | 10 | 3 × 10^3^ |
| *t*_2_ (*t*_1_ < *t*_2_) | Uniform | 3 × 10^3^ | 3 × 10^4^ |
| ***Mutation model*** |  |  |  |
| Mean mutation rate | Uniform | 1 × 10^-6^ | 1 × 10^-3^ |
| Individual locus mutation rate | Gamma | 1 × 10^-7^ | 1 × 10^-2^ |
| Mean coefficient P | Uniform | 1 × 10^-1^ | 9 × 10^-1^ |
| Individual locus coefficient P | Gamma | 1 × 10^-2^ | 9 × 10^-1^ |
