# Supplementary material for: Landscape Features and Climatic Forces Shape the Genetic Structure and Evolutionary History of an Oak Species (Quercus chenii) in East China
Source: Front Plant Sci. 2019 Sep 3;10:1060. doi: 10.3389/fpls.2019.01060 (PMC6734190; doi:10.3389/fpls.2019.01060)
Supplement: Supplementary file 1 [file DataSheet_1.zip › Table_S6.docx]

**Supplementary Table S6** Pearson correlation coefficients among the eight bioclimatic variables and elevation used in this study.

| Variables | Pearson correlation coefficients | | | | | | | | | GLMs | RDA | ENM |
| --- | --- | --- | --- | --- | --- | --- | --- | --- | --- | --- | --- | --- |
|  | elevation | bio1 | bio2 | bio4 | bio8 | bio9 | bio12 | bio15 | bio18 |  |  |  |
| elevation | 1.00 |  |  |  |  |  |  |  |  | * | * | - |
| bio1 | -0.69 | 1.00 |  |  |  |  |  |  |  | * | * | * |
| bio2 | -0.32 | 0.40 | 1.00 |  |  |  |  |  |  | * | * | * |
| bio4 | -0.18 | -0.48 | -0.25 | 1.00 |  |  |  |  |  | * | * | * |
| bio8 | -0.36 | -0.01 | -0.01 | 0.35 | 1.00 |  |  |  |  | * | - | * |
| bio9 | -0.23 | 0.49 | 0.26 | -0.54 | 0.03 | 1.00 |  |  |  | * | * | * |
| bio12 | 0.23 | 0.19 | -0.02 | -0.43 | -0.46 | 0.09 | 1.00 |  |  | * | * | * |
| bio15 | -0.01 | 0.37 | 0.55 | -0.59 | -0.10 | 0.33 | 0.36 | 1.00 |  | * | * | * |
| bio18 | 0.47 | -0.46 | -0.29 | 0.08 | 0.07 | -0.40 | 0.57 | 0.00 | 1.00 | * | * | * |

bio1, annual mean temperature; bio2, mean diurnal temperature range; bio4, temperature seasonality; bio8, mean temperature of wettest quarter; bio9, mean temperature of driest quarter; bio12, annual precipitation; bio15, precipitation seasonality; bio18, precipitation of warmest quarter; *, variables used in general linear models (GLMs), redundancy analysis (RDA) after the forward stepwise selection, and ecological niche modeling (ENM).
