# Supplementary material for: Landscape Features and Climatic Forces Shape the Genetic Structure and Evolutionary History of an Oak Species (Quercus chenii) in East China
Source: Front Plant Sci. 2019 Sep 3;10:1060. doi: 10.3389/fpls.2019.01060 (PMC6734190; doi:10.3389/fpls.2019.01060)
Supplement: Supplementary file 1 [file DataSheet_1.zip › Table_S7.docx]

**Supplementary Table S7** Scores of the eight geographic variables and the eight climatic variables on the first three RDA axes (RDA1–RDA3) in the full RDA model, and both partial RDA models corresponding to pure geography and pure climate. Significance tests based on 999 permutations were performed for each constrained axis and each constraining variable. The proportion of total genetic variation explained by each axis was shown in parentheses.

| Variables | Geography and climate | | | |  | Pure geography | | | |  | Pure climate | | | |
| --- | --- | --- | --- | --- | --- | --- | --- | --- | --- | --- | --- | --- | --- | --- |
|  | *P*-value | RDA1*  (1.78%) | RDA2*  (1.66%) | RDA3*  (1.45%) |  | *P*-value | RDA1*  (1.30%) | RDA2*  (1.18%) | RDA3*  (0.87%) |  | *P*-value | RDA1*  (1.38%) | RDA2*  (1.21%) | RDA3*  (0.80%) |
| PCNM1 | 0.001 | -0.28 | 0.04 | 0.09 |  | 0.001 | 0.10 | 0.25 | -0.06 |  |  |  |  |  |
| PCNM2 | 0.001 | 0.00 | -0.37 | -0.10 |  | 0.001 | -0.19 | 0.08 | -0.30 |  |  |  |  |  |
| PCNM3 | 0.001 | -0.21 | 0.41 | 0.03 |  | 0.001 | -0.02 | -0.21 | -0.18 |  |  |  |  |  |
| PCNM5 | 0.001 | 0.09 | -0.44 | -0.11 |  | 0.001 | 0.26 | 0.17 | 0.18 |  |  |  |  |  |
| PCNM7 | 0.001 | -0.14 | -0.21 | 0.02 |  | 0.001 | 0.27 | 0.15 | 0.16 |  |  |  |  |  |
| PCNM8 | 0.001 | -0.44 | -0.01 | -0.26 |  | 0.001 | 0.07 | -0.09 | 0.03 |  |  |  |  |  |
| PCNM9 | 0.001 | 0.12 | -0.19 | -0.56 |  | 0.001 | -0.14 | 0.47 | 0.17 |  |  |  |  |  |
| PCNM10 | 0.001 | -0.35 | -0.2 | -0.19 |  | 0.001 | 0.68 | 0.02 | -0.59 |  |  |  |  |  |
| elevation | 0.001 | -0.51 | 0.01 | -0.33 |  |  |  |  |  |  | 0.001 | 0.04 | -0.25 | 0.04 |
| bio1 | 0.001 | 0.25 | 0.16 | 0.00 |  |  |  |  |  |  | 0.001 | -0.14 | 0.30 | -0.20 |
| bio2 | 0.001 | -0.17 | -0.12 | 0.40 |  |  |  |  |  |  | 0.001 | 0.38 | 0.12 | 0.15 |
| bio4 | 0.001 | 0.37 | -0.46 | 0.11 |  |  |  |  |  |  | 0.001 | 0.02 | 0.20 | 0.04 |
| bio9 | 0.001 | 0.11 | 0.38 | -0.08 |  |  |  |  |  |  | 0.001 | -0.18 | -0.09 | -0.28 |
| bio12 | 0.001 | -0.15 | -0.30 | -0.20 |  |  |  |  |  |  | 0.001 | 0.08 | 0.15 | -0.06 |
| bio15 | 0.001 | -0.22 | -0.11 | -0.02 |  |  |  |  |  |  | 0.001 | 0.20 | -0.08 | -0.04 |
| bio18 | 0.001 | -0.38 | -0.27 | -0.15 |  |  |  |  |  |  | 0.001 | 0.18 | -0.18 | -0.06 |

bio1, annual mean temperature; bio2, mean diurnal temperature range; bio4, temperature seasonality; bio9, mean temperature of driest quarter; bio12, annual precipitation; bio15, precipitation seasonality; bio18, precipitation of warmest quarter; Eight eigenvectors corresponding to positive eigenvalues of the principal coordinates of neighbor matrix (PCNM) were used as geographic variables. *, *P* < 0.001.
