# Supplementary material for: Landscape Features and Climatic Forces Shape the Genetic Structure and Evolutionary History of an Oak Species (Quercus chenii) in East China
Source: Front Plant Sci. 2019 Sep 3;10:1060. doi: 10.3389/fpls.2019.01060 (PMC6734190; doi:10.3389/fpls.2019.01060)
Supplement: Supplementary file 1 [file DataSheet_1.zip › Table_S8.docx]

**Supplementary Table S8** Probabilities of membership (*Q*) to genetic clusters I–III when *K* = 3 in Bayesian clustering analysis, and *P*-values of heterozygosity excess tests under the stepwise mutation model (SMM) and the two-phase mutation model (TPM) for each population of *Quercus chenii*. Population codes are shown in **Table 1** and **Supplementary Table S1**.

| Population code | *Q*_I_ | *Q*_II_ | *Q*_III_ | *P*-value | |
| --- | --- | --- | --- | --- | --- |
|  |  |  |  | TPM | SMM |
| **Highland populations** | | | | | |
| YZ | 0.94 | 0.04 | 0.02 | 0.52 | 1.00 |
| HS | 0.73 | 0.21 | 0.06 | **0.02** | 0.12 |
| TM | 0.64 | 0.35 | 0.01 | 0.38 | 0.77 |
| QI | 0.04 | 0.93 | 0.03 | 0.52 | 0.97 |
| ZN | 0.93 | 0.01 | 0.06 | 0.27 | 0.94 |
| JZ | 0.04 | 0.94 | 0.02 | 0.85 | 1.00 |
| **Lowland populations** | | | | | |
| XN | 0.28 | 0.18 | 0.55 | 0.20 | 0.95 |
| GD | 0.45 | 0.41 | 0.14 | 0.77 | 1.00 |
| QY | 0.08 | 0.03 | 0.89 | 0.20 | 0.71 |
| WN | 0.27 | 0.15 | 0.58 | 0.75 | 0.99 |
| ZZ | 0.26 | 0.20 | 0.54 | 0.43 | 0.98 |
| WY | 0.01 | 0.21 | 0.78 | 0.67 | 1.00 |
| LU | 0.24 | 0.56 | 0.20 | 0.55 | 0.98 |
| TH | 0.52 | 0.19 | 0.29 | **0.04** | 0.87 |
| LC | 0.33 | 0.40 | 0.27 | 0.21 | 0.79 |
| TY | 0.30 | 0.19 | 0.51 | 0.18 | 0.88 |
| NJ | 0.19 | 0.25 | 0.56 | 0.33 | 0.99 |
| LA | 0.10 | 0.60 | 0.30 | 0.13 | 1.00 |
