# Supplementary material for: Landscape Features and Climatic Forces Shape the Genetic Structure and Evolutionary History of an Oak Species (Quercus chenii) in East China
Source: Front Plant Sci. 2019 Sep 3;10:1060. doi: 10.3389/fpls.2019.01060 (PMC6734190; doi:10.3389/fpls.2019.01060)
Supplement: Supplementary file 1 [file DataSheet_1.zip › Table_S9.docx]

**Supplementary Table S9** Posterior probabilities of the seven scenarios compared in DIYABC.

| Scenario | Posterior probability (95% confidence interval) | |
| --- | --- | --- |
|  | Highland populations | Lowland populations |
| scenario 1 | 0.1424 (0.1375–0.1473) | 0.0854 (0.0815–0.0893) |
| scenario 2 | 0.0159 (0.0132–0.0187) | 0.0096 (0.0069–0.0123) |
| scenario 3 | 0.0483 (0.0454–0.0513) | 0.0152 (0.0126–0.0179) |
| **scenario 4** | **0.3298 (0.3234–0.3361)** | **0.3651 (0.3589–0.3713)** |
| scenario 5 | 0.1514 (0.1465–0.1563) | 0.1927 (0.1875–0.1980) |
| scenario 6 | 0.1415 (0.1369–0.1462) | 0.1583 (0.1535–0.1631) |
| scenario 7 | 0.1706 (0.1660–0.1752) | 0.1736 (0.1691–0.1782) |
