# Supplementary figures and images for: Landscape Features and Climatic Forces Shape the Genetic Structure and Evolutionary History of an Oak Species (Quercus chenii) in East China
Source: Front Plant Sci. 2019 Sep 3;10:1060. doi: 10.3389/fpls.2019.01060 (PMC6734190; doi:10.3389/fpls.2019.01060)

**Supplementary Figure S1** Δ*K* and Ln Pr(*X*|*K*) obtained in Bayesian cluster analysis for each value of *K*.


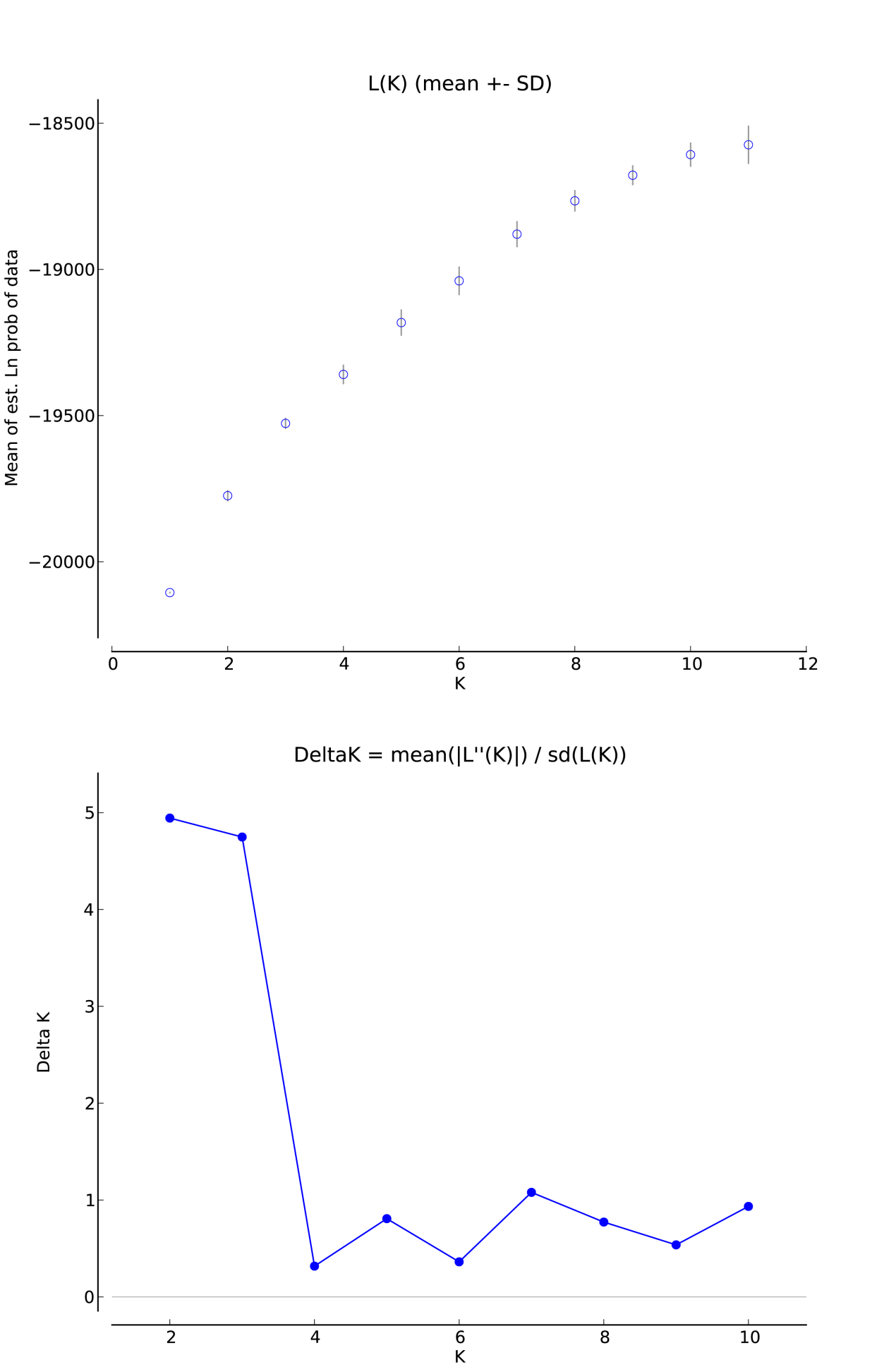

Supplement: Supplementary file 1 [file DataSheet_1.zip › Figure_S1.docx]
